# Mitochondrial targeting by measles virus nucleoprotein modulates viral spread in human airway epithelium

**DOI:** 10.1371/journal.ppat.1013713

**Published:** 2025-11-20

**Authors:** Lorellin A. Durnell-Bettis, Stephanie E. Clark, Camilla E. Hippee, Angela Liu, Justin W. Kaufman, Sydney R. Winecke, Kalpana Yadav, Brajesh K. Singh, Roberto Cattaneo, Patrick L. Sinn

**Affiliations:** 1 Department of Microbiology and Immunology, Carver College of Medicine, The University of Iowa, Iowa City, Iowa, United States of America; 2 Stead Family Department of Pediatrics, Carver College of Medicine, The University of Iowa, Iowa City, Iowa, United States of America; 3 Department of Molecular Medicine, Mayo Clinic, Rochester, Minnesota, United States of America; University of Tokyo Graduate School of Medicine Faculty of Medicine: Tokyo Daigaku Daigakuin Igakukei Kenkyuka Igakubu, JAPAN

## Abstract

Measles is the most infectious human respiratory virus: on average, one individual with measles infects 12–18 susceptible people in a population without immunity. However, how measles virus (MeV) establishes infection in the human respiratory epithelium is insufficiently understood. Since our analyses of MeV infections of well-differentiated primary human airway epithelial cells (HAE) revealed perturbations of mitochondrial gene expression, we tested mitochondrial function. MeV replication disrupted mitochondrial membrane potential and induced superoxide production. This resulted in cGAS-dependent interferon-stimulated gene expression without interferon induction. We then assessed by differential centrifugation whether MeV replicates in mitochondrial proximity. Indeed, MeV proteins and genome were enriched in mitochondrial fractions. We identified a previously unrecognized potential mitochondrial localization signal (MLS) in the MeV nucleoprotein (N), the first protein expressed during infection and showed that the first 70 amino acids of N are sufficient to deliver a GFP reporter to mitochondria. Mutational analyses revealed that arginine 6 and arginine 13 of the N protein are critical for targeting. Recombinant MeV mutants harboring single MLS amino acid substitutions exhibited altered replication kinetics and infectious center formation in HAE, despite similar ISG expression profiles to wild-type MeV. Thus, the MeV N protein amino-terminal arm, previously known only to promote formation of the helical ribonucleocapsid protecting the viral genome, also codes for an MLS. In newly infected cells, this signal may target the formation of MeV replication factories near mitochondria without provoking a canonical RNA sensing pathway. Notably, the MLS appears unique to Morbillivirus N proteins within the Paramyxoviridae family, which are also distinguished by the unique ability to form infectious centers in HAE. Our findings reveal a novel mechanism by which MeV exploits mitochondrial proximity to coordinate replication and modulate host responses, offering new insights into virus-host interactions at the organelle level.

## Author summary

Measles is the most infectious human respiratory virus but the mechanisms favoring its spread in the human respiratory epithelium are incompletely understood. Prompted by our observation of altered mitochondrial gene expression after measles virus (MeV) infection of well-differentiated primary human epithelial cells, we characterized the mechanisms by which MeV interacts with mitochondira in this *ex vivo* system. We discovered that MeV replication centers form near mitochondria and that the virus’s nucleoprotein (N) contains a previously unrecognized mitochondrial localization signal (MLS) in its amino-terminal arm. Mutational analysis revealed that specific arginine residues are essential for mitochondrial targeting and may influence viral replication dynamics. Despite proximity to mitochondria, MeV induces only a limited innate immune response. Our findings provide new insights into how MeV may exploit mitochondrial membranes to facilitate early replication and potentially modulate host immunity and cell-to-cell spread. This work highlights a novel function of the MeV N protein and discloses a new process by which a virus interacts with mitochondria to balance replication and immune evasion.

## Introduction

Measles remains a substantial public health issue. In 2023 it caused >100,000 deaths worldwide, in part, through suppression of host immune functions [1–3]. Measles virus (MeV) is extremely contagious. The CDC estimates that around 9 out of 10 people who are not vaccinated become infected after exposure. Since over 61 million doses of MeV vaccines were postponed or missed during the COVID-19 pandemic, larger outbreaks are now occurring around the world [4–6].

MeV is a negative-strand RNA virus species of the genus *Morbillivirus* in the *Paramyxoviridae* family that includes other severe respiratory pathogens such as Nipah virus, Hendra virus, mumps virus and the parainfluenza viruses [7]. MeV encodes the replicative complex proteins nucleocapsid (N), phosphoprotein (P), and RNA dependent RNA polymerase (L for large) [8]; the membrane fusion apparatus proteins fusion (F) and hemagglutinin (H), whose function is regulated by the matrix (M) protein [7,9–11]; and the innate immunity control proteins C and V [12].

The replication cycle *Morbilliviruses* in their respective hosts differ from that of the other *Paramyxoviridae*. Upon entering a new host, *Morbilliviruses* do not immediately enter and replicate in airway epithelia [7]. Instead, they are picked up by immune cells expressing the signaling lymphocytic activation molecule (SLAM) [13]. These cells deliver infectivity to other SLAM-expressing cells in the local lymph nodes and the primary immune organs, including memory cells. Infection kills many memory cells and weakens immunity to other pathogens, causing immunosuppression [2,14]. Infected immune cells then deliver MeV to airway epithelia expressing the cell adhesion molecule nectin-4 [15].

How MeV replicates and evades host innate immune defenses in airway epithelia is not fully understood. To analyze these processes, we and others rely principally on infection of well-differentiated primary human airway epithelial sheets (HAE). HAE not only recapitulate the multicellular composition of the epithelial layer of the conducting airways but also the innate immune responses to viral, bacterial, and fungal pathogens [16–18].

MeV infects HAE from the basolateral surface and spreads directly cell-to-cell forming infectious centers [19,20]. These infectious centers grow for several days but growth stops before they dislodge *“en masse”* from the epithelial sheet [21]. The number of infected cells in an infectious center varies greatly, but averages ~200 cells [20,22]. The cellular response that stops MeV cell-to-cell spread and defines the size of an infectious center is unknown.

The spread of many respiratory viruses is limited by strong antiviral innate immune responses that include interferon (IFN) production. We recently reported that, in HAE, MeV spread was interrupted by adding exogenous IFN; however, MeV infection did not induce an endogenous IFN mRNA or protein response [22]. In addition, blocking endogenous IFN by receptor antagonists or antibodies had no effect on spread. Despite the lack of induced IFN in MeV-infected HAE, a subset of interferon-stimulated genes (ISGs) was induced [21,22]. In this study, we investigate the source of ISG stimulation in MeV-infected HAE.

The innate immune system evolved to antagonize RNA viruses through pattern recognition receptors (PRRs), such as RIG-like receptors (RIG-I, MDA5, and LGP2). Binding of viral RNA by these sensor proteins elicits downstream antiviral protein expression [23]. DNA viruses are canonically sensed through DNA-sensing-specific PRRs, such as cyclic GMP-AMP synthase (cGAS) [24]. In turn, the cGAS-STING signaling pathway is initiated to perform antiviral functions [25,26]. Multiple studies show that RNA viruses, including MeV, can “non-canonically” activate the cGAS-STING pathway by disrupting mitochondrial integrity and causing mtDNA release [27–34]. Once in the cytoplasm, mtDNA is recognized by cytoplasmic DNA sensors, such as cGAS and IFI16, and drives ISG activation [28,32,35]. These studies reveal an interplay between MeV and mitochondrial function.

Consistent with non-canonical activation of cGAS, we show here that MeV replication in HAE disrupts mitochondrial membrane potential resulting in cGAS dependent ISG expression. MeV proteins and genome were enriched within mitochondrial fractions, suggesting that MeV replication may occur at locations adjacent to mitochondria. In addition, we identify and characterize a mitochondrial localization signal (MLS) in the N protein. These results are consistent with a novel role of the N protein amino-terminal region in localizing MeV replication to the mitochondria, resulting in a mild innate immune response.

## Results

### MeV infection of HAE results in mitochondrial depolarization and superoxide production

**Fig 1:**
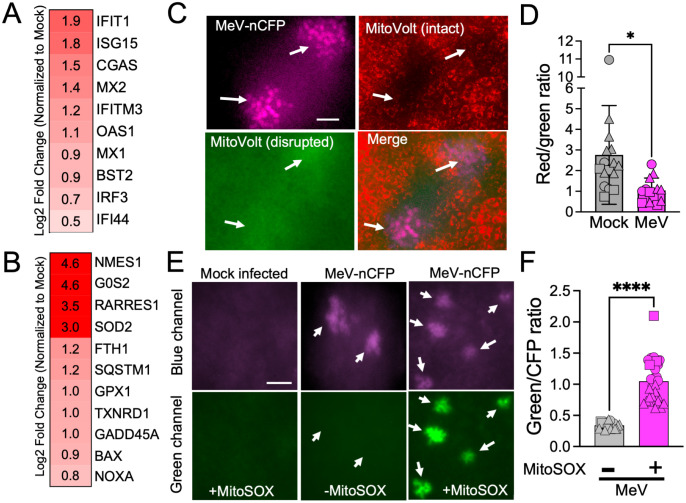
MeV infection damages mitochondria and results in increased levels of superoxide. Heatmaps summarize induced antiviral genes (A) or mitochondrial-related genes (B) in MeV infected HAE. The acquisition of the single-cell RNA sequencing data are previously described^15^. (C) HAE were infected with MeV-nCFP (pseudocolored fuchsia) at MOI 1 for 4 hrs. At 3 dpi, MitoVolt dye was added and mitochondrial membrane potential in individual infectious centers was measured. Arrows point to infectious centers. Scale bar = 50 μm. (D) Red/green ratios indicate mitochondrial membrane integrity. Shapes indicate different donors, n = 3 donors. Each point is one infectious center. (E) HAE were either mock- or MeV-nCFP infected. Cultures were treated with MitoSOX. Superoxide levels were visualized by fluorescent live-cell microscopy. (F) Green/CFP ratios indicated superoxide levels within infectious centers. Shapes indicate different donors, n = 3 donors, scale bar = 50 μm. Each point is one infectious center. *p < 0.05, ****p < 0.0001.

By mining our previously described single-cell RNA sequencing dataset from MeV infected HAE [21], we identified increased mRNAs for 10 archetypal ISGs (Fig 1A) but did not detect canonical IFN signaling [22]. We also observed differential expression of genes related to mitochondrial function (Fig 1B). This suggested that MeV might impact mitochondrial integrity, leading to antiviral gene expression. To visualize the impact of MeV infection on mitochondria, we infected HAE with a MeV expressing nuclear targeted cyan fluorescent protein (nCFP) and visualized mitochondrial membrane potential in live cells 3 days post infection (dpi) using the potentiometric dye, MitoVolt. Membrane potential is reported as the red/green ratio where red fluorescence indicates intact membrane potential and green indicates a loss of potential [36]. As indicated by arrows (Fig 1C), mitochondria within infectious centers were depolarized which led to a significant decrease in the red/green ratio (Fig 1D). These results were recapitulated in H358 cells, an immortalized human airway epithelial cell line (S1A and S1B Fig). In control experiments, we dissipated mitochondrial membrane potential with carbonyl cyanide m-chlorophenylhydrazone (CCCP) and observed the expected response in both HAE (S1C Fig) and H358 cells (S1D Fig).

Because MitoVolt only indicates changes in mitochondrial membrane potential that could result from generalized cellular stress, we next asked if mitochondria are functionally impacted by MeV infection. To this end, we measured levels of superoxide produced within infectious centers using MitoSOX, a live cell probe that fluoresces following oxidation by superoxide. As before, we infected HAE with a MeV-nCFP and applied MitoSOX 3 dpi. Oxidized MitoSOX was readily detected within infectious centers but not uninfected regions (Fig 1E and 1F). No green fluorescence was observed in MeV-nCFP infected cells lacking MitoSOX, confirming that there was no spectrum emission overlap with the CFP channel. These results suggest that MeV induces mitochondrial damage in HAE.

### cGAS inhibition favors MeV spread

**Fig 2:**
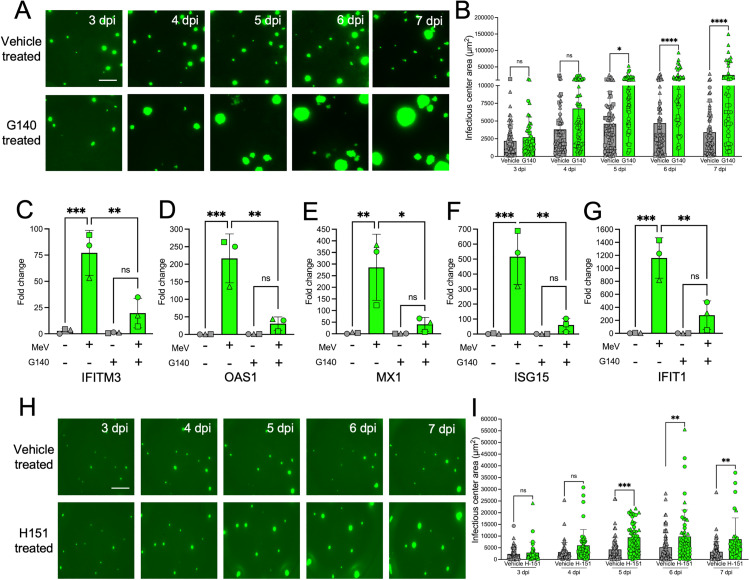
cGAS inhibition favors MeV spread. (A) HAE were infected with MeV-GFP (MOI 1). Immediately after infection, vehicle (DMSO) or G140 (50 μM) was applied basolaterally. Treatments were replaced every 24 hrs. Scale bar = 500 μm. (B) Cells were imaged starting at 3 dpi and infectious center area was measured via ImageJ. (C-G) HAE were mock- (grey) or MeV-infected (green) with or without G140. After 3 dpi, RNA was extracted for ISG expression measurements. (H and I) HAE were infected with MeV-GFP (MOI 1). Immediately after infection, vehicle (DMSO) or H151 (15 μM) was applied basolaterally. Treatments were replaced every 24 hrs. Image capture and infectious center size measurement were performed 3–7 dpi. Scale bar = 500 μm. n = 3 donors for all panels, each shape is a different donor. ns, not significant, *p < 0.05, **p < 0.01, ***p < .001, ****p < 0.0001.

The cGAS-STING pathway is a central response to DNA virus infection [37] but multiple studies also report cGAS-STING activation during RNA virus infection [28–30,32,38,39]. To determine if activation of cGAS impacts MeV infection, we applied a cGAS-specific inhibitor, G140, to the basolateral media. Over the seven day time course, G140 increased cell-to-cell spread of infected cultures as compared to vehicle treatment (Fig 2A and 2B). The mRNA abundance of 5 antiviral genes were quantified by qRT-PCR. IFITM3, OAS1, MX1, ISG15, and IFIT1 mRNA levels were diminished (compared to vehicle treated, MeV-infected HAE) with cGAS inhibitor application in both HAE (Fig 2C–2G) and H358 cells (S2A–S2E Fig).

We inhibited STING with a small molecule inhibitor, H151. H151 treatment of MeV-infected HAE also yielded larger infectious center sizes (Fig 2H and 2I), albeit its effect was less pronounced than that of G140.

In control experiments, we used the cGAS agonist G3-YSD to confirm G140 activity in cells that express endogenous cGAS (i.e., HeLa or H358 cells) or cells without endogenous cGAS expression (i.e., HEK293T cells). We observed a characteristic increase in IFNb mRNA levels following transfection of G3-YSD that was inhibited by the addition of G140 (S2F Fig). G140 increased MeV spread in H358 cells whereas G3-YSD reduced MeV spread (S2G and S2H Fig). G140 had no statistically significant impact on parainfluenza virus 5 (PIV5) or respiratory syncytial virus (RSV) spread in H358 cells (S2I Fig). All together, these results suggest a role for cGAS-STING in inducing ISGs and limiting cell-to-cell spread of MeV in HAE and H358 cells.

**Fig 3:**
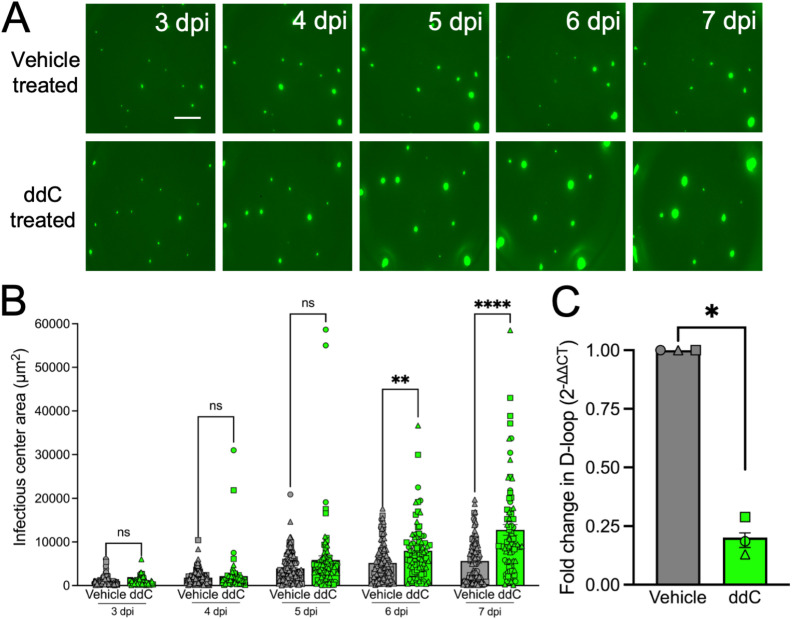
Preventing mtDNA replication increases MeV spread in HAE. (A) 48 hrs pre-infection, HAE were treated with vehicle or ddC (100 μM) in the basolateral media. Treatment was replaced every day during the experiment. 48 hrs post-treatment, HAE were infected with MeV-GFP (MOI 1). Cells were imaged starting at 3 dpi and infectious center areas were measured (B). Scale bar = 500 μm. (C) mtDNA levels were measured using the D-loop gene following vehicle vs ddC treatment to validate mtDNA depletion. n = 3 donors for all panels, each shape is a unique donor. ns, not significant, **p < 0.01, ****p < 0.0001.

If cytosolic mtDNA is the trigger for ISG induction, then depletion of mtDNA by treating cells with a deoxyribonucleoside analogue, 2’,3’-dideoxycytidine (ddC) should result in more cell-to-cell MeV spread and larger infectious centers. Here, HAE were incubated with ddC 48 hrs prior to MeV infection and infectious center size was monitored for 7 dpi. As shown, mtDNA depletion resulted in increased infectious center size in HAE (Fig 3A and 3B) and increased syncytial area in H358 cells (S3A and S3B Fig). mtDNA depletion was confirmed by qPCR of the D-loop mitochondrial gene (Figs 3C and S3C). Viral titers were unaffected by ddC in H358 cells, suggesting that viral replication was not impacted (S3D Fig). However, ISG induction was diminished with ddC treatment in H358 cells (S3E–S3I Fig). These results are consistent with the model that mtDNA mediated activation of cGAS-STING limits MeV cellular spread.

### MeV nucleoprotein contains a mitochondrial localization signal

**Fig 4:**
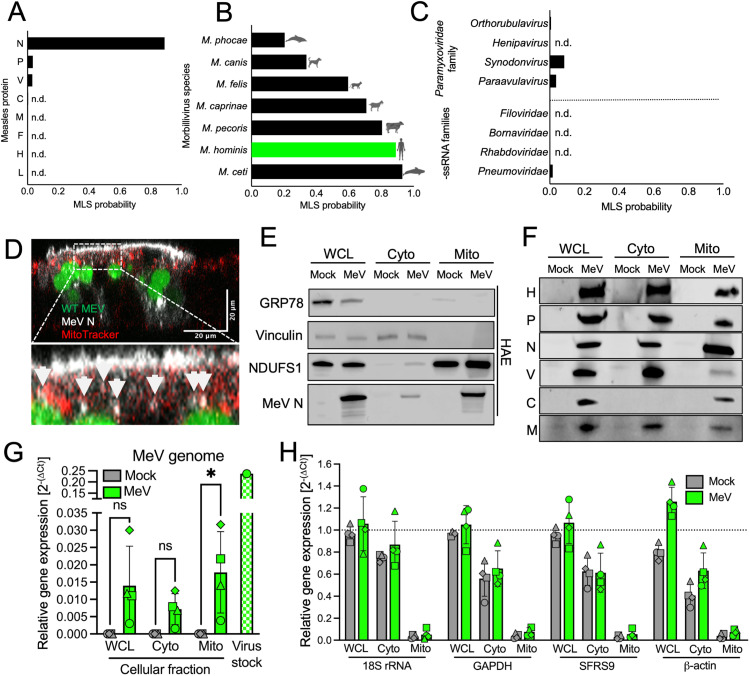
MeV N protein localizes to mitochondria. (A) The MitoFates predicted probability that each MeV protein contains a mitochondrial localization signal (MLS) is plotted. n.d., not detected. (B) The N protein sequences from all seven members of the *Morbillivirus* genus and (C) the N protein sequences from four members outside of the Morbillivirus genus but within the *Paramyxoviridae* family (top) and four members outside of the *Paramyxoviridae* family (bottom) were input into MitoFates. (D) MeV-nYFP (pseudocolored green) infected HAE were co-stained for N protein (white) and MitoTracker (red). Arrows indicate areas of co-localization. (E) HAE were mock- or MeV-infected. WCLs, cytoplasmic, and mitochondrial fractions were isolated and blotted for GRP78 (ER protein), vinculin (cytoplasmic protein), NDUFS1 (mitochondrial protein), and MeV N protein. (F) Western blot showing MeV proteins in the cytoplasmic and mitochondrial fractions of H358 cells. (G) HAE were mock- (grey) or MeV-infected (green). WCLs, cytoplasmic, and mitochondrial fractions were isolated. Virus input stock was used as a positive control. MeV genome levels were measured using primers against intergenic regions of the genome. (H) HAE were mock- or MeV-infected. Cellular fractions were separated, and the RNA was isolated. Levels of 18S rRNA, GAPDH, SFRS9, and β-actin RNA levels were measured. n = 4 donors, each shape is a different donor. ns, not significant; *p < 0.05.

We hypothesized that MeV proteins target mitochondria and induce the observed mitochondrial damage. We used MitoFates, a prediction algorithm, to assess whether any MeV proteins might contain a mitochondrial localization signal (MLS) [40]. Only N was predicted with high probability to contain an MLS (Fig 4A). The N protein from each virus of the *Morbillivirus* genus was predicted with high likelihood to locate to mitochondria (Fig 4B). Members outside of *Morbillivirus* genus but within the *Paramyxoviridae* family are not predicted to contain MLSs (Fig 4C, top). N proteins of negative-strand RNA virus members outside of the *Paramyxoviridae* family, including respiratory syncytial virus, were not predicted to contain an MLS within their N protein sequences (Fig 4C, bottom).

In MeV infected HAE, N protein was observed to co-localize with mitochondria, as indicated by MitoTracker staining (Fig 4D). Using differential centrifugation [41], we isolated whole cell lysate (WCL), cytoplasm, and mitochondria from MeV infected HAE. The presence of N protein or control proteins in each of the fractions was determined by western blot. NDUFS1 is a commonly used mitochondrial marker protein with a known MLS [42]. Vinculin and GRP78 are two proteins known to be associated with the cytoplasm and endoplasmic reticulum [43], respectively. All proteins were detected in the WCL, but only NDUFS1 and MeV N protein were readily detected in the mitochondrial fraction (Fig 4E). This result was recapitulated in H358 cells (S4A Fig) and primary monocyte derived human dendritic cells (S4C–S4E Fig). The lack of GRP78 in the cytoplasm suggests that the endoplasmic reticulum is removed as part of the centrifugation process. Five other MeV proteins, in addition to N, were also detected in the mitochondrial fraction of infected H358 cells (Fig 4F). Of note, MitoFates appropriately predicts NDUFS1 to locate to the mitochondria but not vinculin (S4B Fig).

Since the N protein encapsidates the MeV genome, we assayed for viral genomes in fractionated cell extracts of MeV infected HAE using qRT-PCR. MeV was detected in both mitochondrial and cytoplasmic fractions using genome specific amplification primers (Fig 4G). Genome detection in the mitochondrial fraction was not due to RNA contamination as suggested by the reduced levels of 18S rRNA, GAPDH, SFRS9, and β-actin mRNA in the mitochondrial fraction as compared to the WCL and cytoplasmic fractions (Fig 4H). Preferential representation of MeV proteins and genomes in the mitochondrial fraction suggests that MeV replication occurs in the proximity of mitochondria.

### N protein MLS delivers GFP to mitochondria

**Fig 5:**
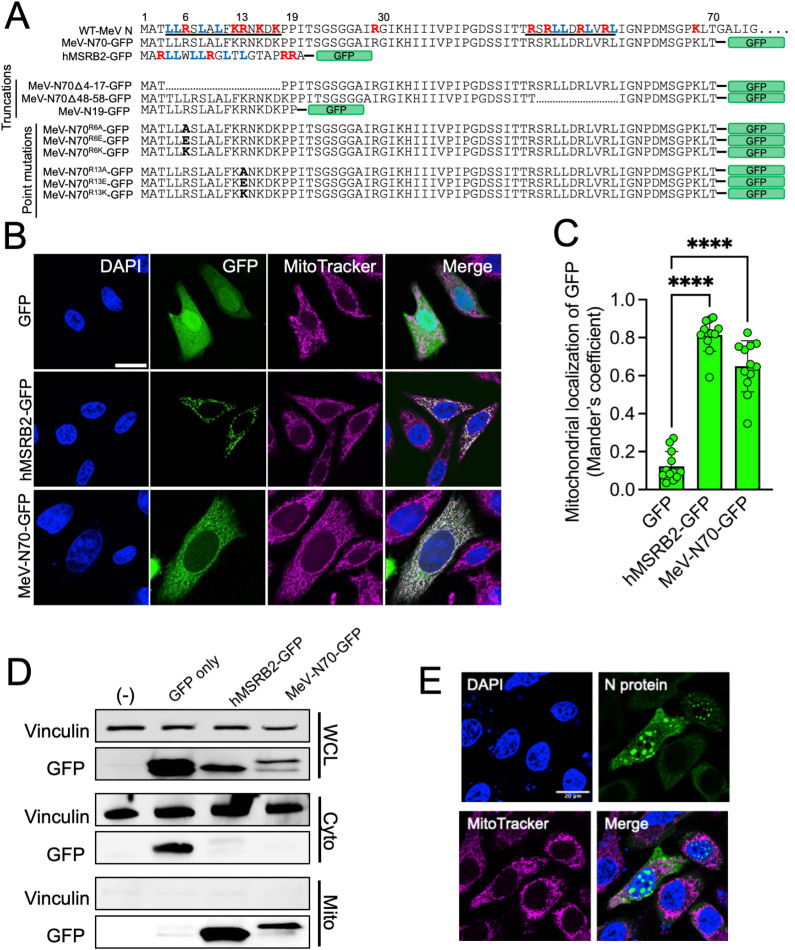
The MLS of MeV N protein delivers GFP to mitochondria. (A) Sequence alignments of plasmid constructs depicting WT and mutated sequences. Red indicates arginine and lysine residues. Blue indicates leucine residues. Bold residues indicate point mutations. Underlined regions indicate putative MLS functional domains. (B) Plasmids expressing GFP only, hMSRB2-GFP, or MeV-N70-GFP were transfected into HeLa cells. Cells were stained with MitoTracker followed by fixation and DAPI staining. Scale bar = 50 μm. (C) Mander’s coefficient analysis of co-localization observed in B is shown. Each point indicates one cell from 3 independent experiments. n = 12 cells. (D) Untransfected (-), GFP only, hMSRB2-GFP, or MeV-N70-GFP transfected HeLa cells were subjected to cytoplasmic and mitochondrial isolation followed by western blot against vinculin and GFP. (E) HeLa cells were transfected with a GFP tagged full length N protein expression plasmid. 18 hrs post-transfection, cells were treated with MitoTracker, fixed, and counterstained with DAPI. ****p < 0.0001.

Next, we investigated the ability of the N-terminus of MeV N protein to direct a heterologous protein to mitochondria. We cloned the first 70 amino acids of N upstream of green fluorescent protein (GFP). This expression plasmid was termed MeV-N70-GFP (Fig 5A). As a control, a matched plasmid using the MLS (the first 20 amino acids) of human methionine-R-sulfoxide reductase B2 (hMSRB2) [44], termed hMSRB2-GFP, was generated. HeLa cells were transfected with MeV-N70-GFP, hMSRB2-GFP, or a GFP-only expressing plasmid and labeled with MitoTracker (Fig 5B). As expected, GFP-only transfected HeLa cells showed even cytoplasmic distribution of GFP. However, both hMSRB2-GFP and MeV-N70-GFP significantly co-localized with mitochondria as determined by Mander’s coefficients (Fig 5C).

To complement these analyses, we isolated the cytoplasmic and mitochondrial fractions from un-transfected, GFP-only, hMSRB2-GFP, and MeV-N70-GFP transfected HeLa cells (Fig 5D). Similar amounts of the vinculin loading control were detected in the WCL and cytoplasmic fractions of all transfection samples. The GFP proteins from the hMSRB2-GFP and MeV-N70-GFP transfected cells were readily detected in the mitochondrial fraction but not the cytoplasmic fraction.

We also note that transfection of a plasmid expressing full-length N results in punctate nuclear and cytoplasmic localization (Fig 5E). This is consistent with previous observations that full length N contains a cryptic nuclear localization signal and accumulates in the nucleus unless retained in the cytoplasm by P protein co-expression [45]. Of note, in bona fide MeV infection of HAE, N preferentially localizes on actin rings proximal to the apical plasma membrane [46]. This is where mitochondria accumulate in well-differentiated airway epithelial cells [47].

### Functional analysis of the N protein MLS

**Fig 6:**
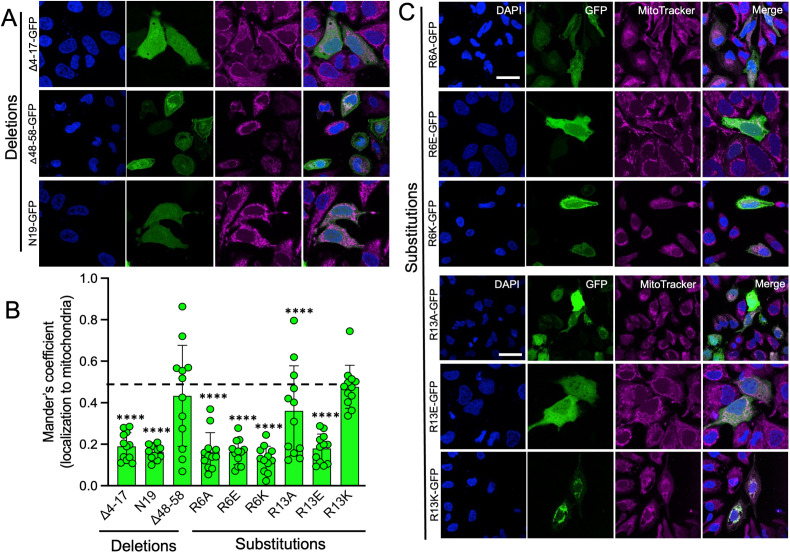
Mutated MeV N protein MLSs fail to deliver GFP to mitochondria. (A) HeLa cells were transfected with the indicated N-protein deletion mutant expression plasmids. 18 hrs post-transfection, cells were stained with MitoTracker, fixed, and counterstained with DAPI. (B) Mander’s coefficient analysis of transfected cells. Dashed line indicates N70-GFP. (C) HeLa cells were transfected with the indicated N-protein point mutant expression plasmids. 18 hrs post-transfection, cells were stained with MitoTracker, fixed, and counterstained with DAPI. n = 12 cells per transfection. ****p < 0.0001 vs N70.

MLS domains tend to be enriched in arginine, lysine, and leucine residues (red and blue amino acids, Fig 5A) that fold into amphipathic helixes [48]. Such structures allow for interaction with the protein complexes crucial for mitochondrial protein import [49]. We asked if specific amino acids or motifs were necessary for mitochondrial targeting. Two putative MLS domains (positions 4–17 and 48–58) were identified based on arginine, lysine, and leucine clustering. As shown schematically (Fig 5A), deletion mutants were generated (MeV-N70D4-17-GFP and MeV-N70D48-58-GFP) along with a minimal putative MLS containing only the initial 19 amino acids of N (MeV-N19-GFP). Following transfection into HeLa cells and co-labeling with MitoTracker (Fig 6A and 6B), we observed that deletion of positions 4–17 ablated mitochondrial targeting. The initial 19 amino acids were also unable to re-localize GFP (Fig 6A and 6B). Deletion of positions 48–58 had minimal impact on mitochondrial targeting. These results suggest that the first arginine/lysine/leucine rich motif is necessary but insufficient for maximal MLS activity.

As shown schematically in Fig 5A, we next focused on arginine (R) at positions 6 and 13 to assess whether minimal modifications impact MLS function. Mutation to alanine (A) converts a positively charged side chain to a small, neutral side chain; mutation to glutamic acid (E) converts to a negative side chain; and mutation to lysine (K) converts to a different positively charged side chain. MitoFates predicted that R6E would have the greatest reduction in the probability of MLS activity, R6K would have minimal reduction, and R6A would be intermediate (S5 Fig). In each case, modification of R13 was predicted to be less impactful than R6. The experimental results were largely consistent with the MitoFates predictions (Fig 6B and 6C). Amino acid substitutions R6A, R6E, R13A, and R13E resulted in significantly reduced GFP localization to mitochondria, whereas, R13K had little impact on mitochondrial localization. Unlike the MitoFates prediction, R6K resulted in loss of GFP localization to mitochondria.

### N protein mutations alter MeV infection of HAE

**Fig 7:**
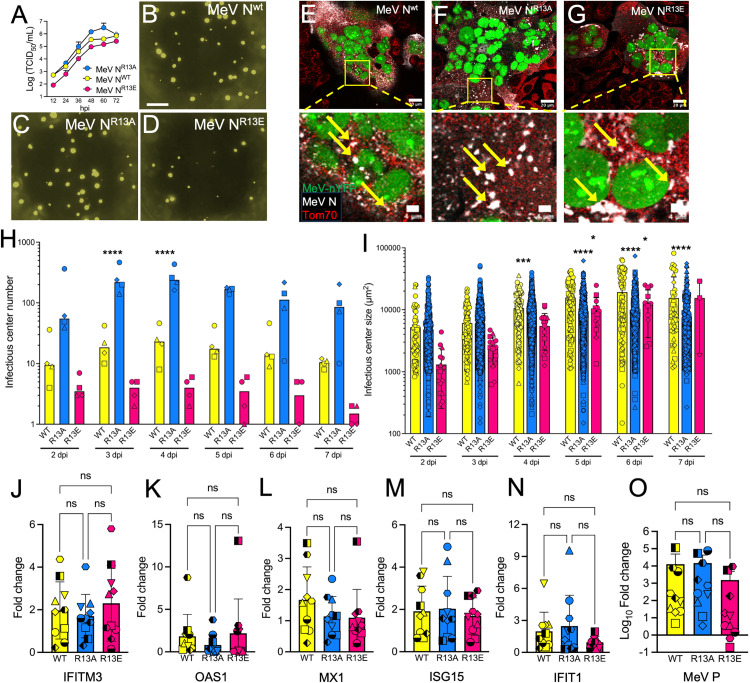
Mutation of MeV N alters infection pattern in HAE. (A) Growth kinetics for the parental virus (MeV N^WT^) expressing nYFP and two derivatives (MeV N^R13A^ and MeV N^R13E^) in HeLa hSLAM cells infected at an MOI of 0.05. Cell lysates were collected every 12 h and titrated on Vero hSLAM cells. (B-D) HAE were infected with the indicated virus and imaged at 5 dpi. Scale bar = 1 mm. (E-G) High powered images of H358 cells were infected with the indicated virus co-stained for N protein (white) and Tom70 (red). Arrows indicate areas of co-localization. HAE were infected at an MOI of 0.1 and imaged daily from 3-7 dpi. Infectious center number (H) and size (I) were measured via ImageJ. (J-N) After 3 dpi, RNA was extracted for ISG expression analysis or (O) MeV P mRNA expression analysis (MOI = 0.1). n = 3 donors for H and I; n = 10 donors for panels J-O, each filled or half-filled shape is a unique donor. ns, not significant, *p < 0.05, **p < 0.01, ***p < 0.001, p < 0.0001.

To determine the consequence of mutating the MLS of the N protein for MeV replication, we sought to engineer viruses with mutations in R6 and R13. However, we were only able to rescue viruses with amino acid substitutions at position 13, which we termed MeV-N^R13A^ and Mev-N^R13E^. These viruses had similar growth curves as the parental MeV-N^WT^ on HeLa-hSLAM cells (Fig 7A). Each virus expressed a nuclear targeted (n)YFP reporter gene and formed infectious centers in HAE (Fig 7B–7D). Confocal microscopy analyses performed at 5 dpi indicated that the N proteins from WT MeV, MeV-N^R13A^, and MeV-N^R13E^ all displayed at least partial colocalization with Tom70, a marker for the mitochondrial outer membrane (Figs 7E–7G, S6A and S6B). Incomplete detargeting of MeV-N^R13A^ and Mev-N^R13E^ from mitochondria is consistent with MitoFates predictions (S5 Fig).

As compared to MeV-N^WT^, MeV-N^R13A^ infection resulted in more numerous infectious centers; whereas, MeV-N^R13E^ resulted in fewer infectious centers (Fig 7H). As compared to MeV-N^WT^, infectious center growth (an indicator of cell-to-cell spread kinetics) was more rapid for MeV-N^R13A^ and slower for Mev-N^R13E^; however, the average infectious center size reached the same plateau for all three viruses by 5 dpi (Fig 7I). The mRNA abundance of 5 antiviral genes was quantified by qRT-PCR. IFITM3, OAS1, MX1, ISG15, and IFIT1 mRNA levels were similar for all 3 viruses in HAE (Fig 7J–7N). At 3 dpi, the abundance of the 3 viruses in HAE was not significantly different as indicated by MeV P mRNA levels (Fig 7O). These results indicate that mutations in the N-protein MLS domain impact MeV cellular spread in HAE without overt changes in ISG activation.

## Discussion

Mitochondria are dynamic organelles vital for energy production that also play a crucial role in the innate immune response. During microbial infections, their outer membrane serves as signaling hub to induce innate immune responses that counteract invading pathogens like viruses [50]. MeV infection can downregulate mitochondrial biogenesis, activate cGAS-STING, and induce mitofusion-1-mediated mitochondrial fusion [28]. However, it was not known how MeV interacts with mitochondria. We report here that, in a well-differentiated primary culture model of human airway epithelial cells, it sets up replication centers in the proximity of mitochondria. We identified an MLS in the N protein and showed that it can deliver a reporter protein to the mitochondria. Mutational analyses indicated that two arginines (positions 6 and 13) were both important for mitochondrial localization of GFP. We attempted to generate viruses with mutations at both arginines but only MeV-N^R13A^ and MeV-N^R13E^ replicated efficiently. Thus, arginine 6 may be essential for replication.

An unexpected result of these studies was the phenotypic differences between MeV-N^R13A^ and MeV-N^R13E^. Based on MitoFates predictions and on transient expression analyses of the mutated MLS-GFP protein hybrids localization, we anticipated partial mitochondrial de-targeting for MeV-N^R13A^ and MeV-N^R13E^, but confocal microscopy analyses indicated similar mitochondrial co-localization for both mutants to MeV-N^wt^. HAE infection experiments revealed that MeV-N^R13A^ initially produced more infectious centers than the parental virus, while MeV-N^R13E^ produced less. Infectious center growth was initially more rapid for MeV-N^R13A^ than for the parental virus, and slower for MeV-N^R13E^. On the other hand, the infectious center size reached the same plateau after 5 days for all three viruses. Moreover, the mRNA abundance of five antiviral genes was similar in all infections. We are currently generating additional substitutions to further probe this phenomenon in future studies. These studies will investigate if N protein amino acid substitutions impact viral replication, transcription, protein/protein, and protein/RNA interactions within airway cells.

The primary function of the N protein is to encapsidate viral genomic RNA, forming a helical structure that protects it from host endonuclease-mediated degradation [8]. Atomic level structural analyses of the arrangement of the N protein subunits in this ribonucleocapsid revealed that the amino-terminal 36 amino acids of each subunit form an “arm” that extends away from the central “core” domain and contacts the core domain of a neighboring subunit [51]. In particular, the first 14 amino acids of this arm folds into an alpha-helical structure. This alpha-helical structure overlaps almost entirely with the first motif of the N MLS, predicted to extend from position 4–16 and includes arginines 6 and 13. AlphaFold software predicted that the alpha-helical structure was retained for the R13A and R13E amino acid substitutions as well as the R6A and R6E substitutions.

How would the newly discovered N protein MLS facilitate MeV replication? An MLS is recognized by receptor proteins on the outer mitochondrial membrane, typically part of the translocase of the outer membrane (TOM) complex [52]. We postulate that during protein synthesis the N MLS may interact with mitochondrial translocases. These interactions would concentrate the N proteins, the first viral gene products expressed in an infected cell, on a mitochondrial membrane platform. Local concentration of N proteins on a membrane may facilitate helical nucleocapsid formation around nascent MeV genomic RNA. In this process N protein amino-terminal arms would transfer from their cellular protein interactor to other N proteins.

Notably, the mitochondrial antiviral signaling (MAVS) complexes are also located in the outer mitochondrial membrane. We do not know how MeV escapes immune detection, nor do we know why cGAS activation fails to lead to IFN activation. We note that the MeV P gene codes for two innate immunity control proteins named C and V [12,53] that control immune evasion. It was recently reported that an attenuated MeV vaccine strain initially induces an increase in mitochondrial respiration and ATP production, likely leading to enhanced viral replication [35]. Eventually, however, infection causes degradation of the mitochondrial network. Of note, V contributes to these processes [35]. We speculate that mitochondrial-proximal seeding of MeV replication sites by the N MLS could facilitate enhanced respiration activity, and the eventual degradation of the mitochondrial network may ultimately contribute to constraining the infectious center growth observed in HAE.

Several other RNA viruses impact mitochondrial functions [50]. Positive strand flaviviruses alter mitochondrial processes to evade the innate immune response [54]. Positive-strand norovirus NS3 protein, which has a mitochondrial localization signal, initiates cell death to induce viral egress [38,55]. The tripartite negative-strand Rift Valley Fever Virus, which can cause fatal hemorrhagic fever, targets its NSm protein to the mitochondrial outer membrane and exerts anti-apoptotic function [56]. The multipartite negative-strand influenza virus targets its PB1-F2 protein to mitochondria [57,58], and can alter mitochondrial morphodynamics and the innate immunity signaling RIG-I complex at mitochondria [59].

Of note, we observe little cytopathic effect following MeV infection of HAE [21]. Perhaps by locating to mitochondria, N protein may favor cell survival. Indeed, Epstein-Barr virus BHRF1, human herpes virus 8 vBCL-2, and adenovirus E1B19K, amongst others can promote cell survival by locating to mitochondria and interfering with the function of host mitochondrial proteins, Bax/Bak, that normally regulate pro-apoptotic signals [60–68]. In MeV infections of HAE, we previously noted that SK1-dependent live-cell extrusion occurs and explains, in part, why cells detach from the epithelial layer [21,69]. Sphingosine-1-phosphate, the product of SK1, has been shown to be associated with cell-survival [70,71].

Interestingly, only the N proteins of the members of the *Morbillivirus* genus within the *Paramyxoviridae* family are predicted to code for an MLS. Morbilliviruses are the only dual tropic *Paramyxoviridae*, having acquired the capacity to replicate efficiently not only in epithelial cells but also in lymphocytic cells [7]. Our observation that MeV mitochondrial targeting also occurs in monocyte-derived human dendritic cells suggests that N protein directed mitochondrial proximal replication is also relevant in immune cells.

In conclusion, we discovered that the amino-terminal arm of the MeV N protein, whose previously recognized function is to promote the formation of a helical ribonucleocapsid protecting the viral genome, also codes for a mitochondrial localization signal. We propose that, in newly infected cells, early localization of N proteins near mitochondria may promote the local development of MeV replication sites [72,73] and local mitigation of non-canonical cGAS-STING pathways. Further studies are required to define the relationships between mitochondrial targeting, ISG expression, and the cessation of infectious center growth.

## Methods

### Ethical statement

The University of Iowa *In Vitro* Models and Cell Culture Core prepared the well-differentiated primary cultures of human airway epithelia (HAE) used in these studies. These cultures are comprised of cells from autopsy, discarded tissue, or surgical specimens. All human subject studies were conducted with approval from the University of Iowa Institutional Review Board. We do not receive identifiable information concerning tissue donors and informed consent is not applicable.

### Cells

HAE were grown and maintained by the University of Iowa’s *In Vitro* Models and Cell Culture Core. Briefly, epithelial cells were enzymatically dissociated from tracheal and bronchial tissue as previously described [16]. Isolated cells were then seeded onto collagen-coated polycarbonate costar transwells (0.4 µm pore size, surface area of 0.6 cm^2^; Corning Costar, Cambridge, MA). The cells were incubated at 37°C, 5% CO_2_ at an air-liquid interface in Ultroser G (USG). The cells were allowed to differentiate for at least three weeks post seeding. H358 cells (bronchioalveolar carcinoma cells line, ATCC cat# CRL-5807) were grown in Roswell Park Memorial Institute 1640 medium (RPM1 1640, ThermoFisher Scientific) supplemented with 10% fetal bovine serum, amphotericin-B (0.25 µg/mL final concentration), and 50 µg/mL-50 U/mL penicillin-streptomycin. HEK293T cells (ATCC cat# CRL-3216) were grown and maintained in RPM1 1640 medium supplemented with 10% fetal bovine serum, amphotericin-B (0.25 µg/mL final concentration), and 50 µg/mL-50 U/mL penicillin-streptomycin. HeLa cells (ATCC cat# CCL-2) were grown and maintained in Dulbecco’s Modified Eagle Medium (DMEM, ThermoFisher) supplemented with 10% fetal bovine serum, amphotericin-B (0.25 µg/mL final concentration), and 50 µg/mL-50 U/mL penicillin-streptomycin. Vero cells expressing the human Signaling Lymphocyte Activation Molecule (SLAM) (Vero-hSLAM) were grown and maintained in DMEM supplemented with 10% fetal bovine serum, amphotericin-B (0.25 µg/mL final concentration), 50 µg/mL-50 U/mL penicillin-streptomycin, and 500 µg/mL G418-geneticin.

### Virus rescue, stock production, and growth kinetics

Infectious MeV (IC323, Ichinose-B isolate) recombinantly expressing cytoplasmic green fluorescent protein (GFP), nuclear targeted cyan fluorescent protein (nCFP), or nuclear targeted yellow fluorescent protein (nYFP) were cloned, rescued, and produced as previously described [74,75]. Deep sequencing was used to confirm the identity of previously established and newly established virus stocks as described previously [76]. To propagate, Vero-hSLAM cells were infected at an MOI of 0.01 in serum-free medium for 2 hours. After infection, cells were incubated in Dulbecco’s high-glucose modified Eagle’s medium (DMEM; ThermoFisher Scientific) supplemented with 10% fetal bovine serum (FBS), 0.25 μg/mL amphotericin-B, 50 μg/mL penicillin, 50 U/mL streptomycin, and 500 μg/mL G418-geneticin [77]. Two dpi, cell lysates were collected and subjected to 3 rounds of freeze-thaw cycles. Supernatants were clarified with centrifugation and aliquoted for storage. For growth kinetic analyses, HeLa-hSLAM cells were seeded in 6-well plates at a density of 2.5x10^5^ cells per well. The next day, cells were infected with passage 1 stocks at an MOI of 0.05. Supernatant and cell lysates were collected every 12 hours for up to 72 hours post-infection (hpi). Supernatants were centrifuged to clear debris before freezing at -80°C. Cell lysates collected in 500 µl Opti-MEM were subjected to three freeze thaw cycles. Viral titrations were determined via standard TCID_50_/mL assay on Vero-hSLAM cells [12].

Recombinant respiratory syncytial virus strain A2 expressing GFP reporter (RSV-GFP) was provided by Mark Peeples [78] and produced as previously described [22]. Recombinant parainfluenza virus 5 expressing mCherry reporter (PIV5-mCherry) [79] propagation and titer methods are available upon request.

### Infection of HAE

HAE cultures in this study were infected with MeV as previously published [19,80]. Briefly, HAE cultures were inverted to expose the basolateral surface. 50 μL virus inoculum, containing MeV virus and serum-free opti-MEM (Thermo Fisher Scientific; #31985062) was placed on the basolateral side of the cells. Virus inoculum was incubated on HAE for ~4 hours at 37°C and 5% CO_2_. After the infection period, the inoculum was removed and the cultures were placed back into an upright position with medium in the basolateral chamber.

### Inhibitor treatments

H358 and HAE cultures were infected, and inhibitor treatments were immediately added to cell medias. All inhibitor treatments on HAE were applied to the basolateral chamber in USG medium. Treatments were replaced every 24 hours until the end of the experiment. G140 (InvivoGen, cat# inh-g140) was applied at final working concentration of 50 μM. in HAE and 10 μM in H358 cells H151 (InvivoGen, cat# inh-h151) was applied at a final working concentration of 15 μM. The cGAS agonist G3-YSD (InvivoGen, cat#tlrl-ydna) was transfected (3 µg/ml) into HEK293T, H358, or HeLa cells with Lipofectamine 2000 following the manufacturer’s protocol. After 48 hours, cells were harvested with TRIzol, mRNA was extracted, and IFN-β transcripts were measured via qRT-PCR. Fluorescence was quantified using ImageJ.

### Mitochondrial depletion assay

HAE and H358 cultures were treated with ddC (2’,3’-Dideoxycytidine hydrochloride) (Millipore, cat# D0776) 48 hours prior to infection and replaced every day before and after infection. Treatments were applied to the basolateral medium for HAE cultures. For H358 cells, ddC was maintained in culture during the entire experiment. The final concentration of ddC was 100 μM for all experiments.

### Mitochondrial membrane potential measurement and analyses

Mitochondrial membrane potential was measured using a JC-10 kit, ION VITAL-MitoVolt (Ion Biosciences, cat# 5100). Reagent was prepared following manufacturer’s instructions and applied to cells for 30 minutes. For HAE, reagent was applied both apically and basolaterally. After incubation, cultures were live-cell imaged. Red/green ratios were assessed by using fluorescence intensity measurements output by ImageJ. To measure membrane potential of infectious centers, the area around the infectious center was traced and saved. Saved tracings were applied to the green and red channels for each respective infectious center and measurements were taken. Mock-infected measurements were made using similar area tracings. Red/green ratios for H358 cells were assessed by measuring the fluorescence intensity of entire field of focus. To induce mitochondrial membrane potential depolarization, carbonyl cyanide m-chlorophenylhydrazone (CCCP) (ThermoFisher, cat# L06932.ME) was applied to cultures for 30 minutes prior to MitoVolt treatment and live-cell microscopy.

### Superoxide measurement and analysis

HAE were either mock- or MeV-nCFP-infected. After 3 dpi, cells were incubated with mitoSOX green as per manufacturer’s guidelines (ThermoFisher, cat# M36008). Live images were captured, and the superoxide fluorescence intensity was divided by the virus fluorescence intensity output by ImageJ.

### Cytoplasm and mitochondria fractionations

Fractionation was performed based on Dixit, et al. [41]. Briefly, cells were washed 1x with 1X DPBS. TrypLE was added to cells (ThermoFisher, cat# 12604021) and incubated at 37°C for 10–15 minutes. Cells were collected and pelleted followed by a wash step with 1X DPBS. Washed pellet was resuspended in 1X hypotonic buffer (50 mM HEPES, 1 mM EDTA, 1 mM Dithiothreitol, 1X protease inhibitor cocktail (Roche, cat# 11836170001), 1 mM phenylmethanesulfonylfluoride, 1 mg/mL bovine serum albumin), incubated on ice for 5 minutes. High purity digitonin (Millipore Sigma, cat# 11024-24-1) was added and the cells were vortexed vigorously every minute up to 5 minutes total. An equal volume of 2X isotonic buffer (100 mM HEPES, 2 mM EDTA, 2 mM dithiothreitol, 1X protease inhibitor cocktail, 2 mM phenylmethanesulfonylfluoride, 2 mg/mL bovine serum albumin, 1.2 M D-Sorbitol) was added to the cell suspensions, diluting it to 1X prior to centrifugation at 700 x g for 5 minutes. The crude pellet was discarded while the supernatant was centrifuged again at 10,000 x g for 15 minutes at 4°C. The supernatant was collected and labelled cytoplasmic fraction. The pellet was washed once with 1X isotonic buffer followed by another centrifugation at 10,000 x g for 10 minutes at 4°C. The supernatant was removed. For genome analyses, this pellet was immediately put into TRIzol for RNA extraction. For western blot analyses, RIPA Lysis and Extraction Buffer (ThermoFisher Scientific cat# 89900) containing a cOmplete, Mini EDTA-free Protease Inhibitor Cocktail tablet (Millipore Sigma, Darmstadt, Germany cat# 11836170001) was added and prepped for western blot. WCLs were prepared in parallel, lysed with RIPA buffer containing protease inhibitors, and not subjected to fractionation.

### Transfection assays

Cells were transfected with Lipofectamine 2000 following manufacturer’s instructions (ThermoFisher, cat# 11668019) followed by TRIzol collection 18 hours post-transfection for RNA isolation. For MeV N70-GFP localization experiments, including control and mutant plasmids, HeLa cells were plated on coverslips and transfected with Lipofectamine 2000. 18 hours post-transfection, cells were treated with MitoTracker (ThermoFisher, catalog # M7512) prior to fixation. For N protein localization, plasmids expressing full-length N protein were transfected into HeLa cells with Lipofectamine 2000. 18 hours post-transfection, cells were stained with anti-N_505_ antibody to detect N protein.

### Immunostaining and confocal microscopy

All cells prepaired for immunofluorescence were fixed with 4% paraformaldehyde for 15 min followed by washing with 1X PBS and permeabilization with 0.1% Triton X-100 in Superblock (ThermoFisher Scientific; #37580) for 1 h at RT. Cells were treated with MitoTracker (ThermoFisher, catalog # M7512) prior to fixation. Primary antibodies were incubated in Superblock overnight at 4˚C. Cells were then washed 3x with 1X PBS and incubated with fluorescently labeled secondary antibodies and counterstains in Superblock buffer for 1h at RT. HAE filters were cut from transwells, mounted on glass microscopy slides with VECTASHIELD Mounting Medium (Vector Laboratories; #H100010), and covered with a glass coverslip. Primary antibodies and dilutions used: MeV N 1:500 (Sigma Aldrich; #MAB8906), TOM70 (Proteintech; #CL594–14528, Rabbit polyclonal, 1:200 dilution). Secondary antibodies and counterstains used: F-actin Phalloidin Alexa Fluor Plus 647 1:400 (Invitrogen; #A30107), F-actin Phalloidin Alexa Fluor Plus 405 1:400 (Invitrogen; #A22281), goat anti-mouse 647 1:1000. Images were collected using a Leica SP8 (Leica Microsystems) or Olympus confocal microscopes and quantified in ImageJ. Low power or live cell images were collected using a Keyence BZ-Z8100 (Keyence Corporation of American, Itasca. IL, USA).

### SDS-PAGE and western blot

Laemmli buffer was added to WCL, mitochondrial- and cytoplasmic-fractions, and each sample was boiled at 95°C for 5 min. Samples were loaded into a 4%–20% Mini-PROTEAN TGX Precast Protein Gel (BioRad, Hercules, CA cat# 4561094) and ran at 100 V for 30–45 min. Gels were transferred onto PVDF membranes at 25 mA overnight at 4°C. Membranes were blocked with 1X TBS buffer supplemented with 0.1% Tween and 5% milk for 1 hour at room temperature. Primary monoclonal and polyclonal antibodies used for assessment are as follows: polyclonal rabbit NDUFS1 (Cell Signaling Technology, cat# 70264), monoclonal rabbit anti-vinculin (ThermoFisher Scientific cat# 700062), polyclonal rabbit anti-N_505_, monoclonal mouse GRP78 (Invitrogen, cat# MA5–27686). Antibody dilution for all primary antibody incubations was 1:1,000. For the secondary antibody, horseradish peroxidase-conjugated goat anti-rabbit or anti-mouse (Millipore Sigma cat# 111-035-144, ThermoFisher Scientific cat# 31430) was used at 1:10,000 dilution for 1 hour. Blots were developed with SuperSignal West Pico PLUS Chemiluminescent Substrate (ThermoFisher Scientific cat# 34580).

### MitoFates

The first 100 N-terminal amino-acids of all indicated proteins were analyzed by MitoFates. All non-virus protein sequences were obtained using the NCBI protein database (https://www.ncbi.nlm.nih.gov/protein/). All virus protein sequences were obtained from the NCBI virus data base (https://www.ncbi.nlm.nih.gov/labs/virus/vssi/#/). Accession numbers for proteins used for analysis can be found in S1 and S2 Tables. FASTA sequences were entered into MitoFates [40] and the output was recorded. For MitoFates analyses and plasmid cloning (discussed below), sequence from the IC323 isolate was used; however, a MitoFates analysis of 7 different MeV isolates (including vaccine strains) showed identical MLS probabilities (S1 Table).

### Cloning

MeV-N70-GFP and hMSRB2-GFP were designed *in silico* using SnapGene and synthesized by Genscript into the pcDNA3.1 GFP plasmid. Following plasmid acquisition, DH5α bacterial cells were transformed and plated. Colonies were picked the next day. Luria broth cultures were inoculated and shaken overnight at 37°C, shaking at 250 RPM. The next morning, the bacterial cultures were subjected to DNA isolation, glycerol stock generation, and DNA sequencing to confirm successful transformation and plasmid recovery. For generation of mutants, primers were harboring mutations or truncations were developed with the NEBaseChanger tool. Site-Directed Mutagenesis was performed following manufacturers guidelines (NEB, catalog # E0553). Transformations were performed as described above. Sequence mutations were confirmed via sequencing. Primers for mutagenesis reactions can be found in S3 Table.

### Colocalization assays and Mander’s coefficient determinations

HeLa cells were transfected as described above. After MitoTracker treatment (ThermoFisher, cat# M7512), transfected HeLa cells were fixed with 4% paraformaldehyde (PFA) for 15 minutes. Cells were washed 3x with 1X DPBS in 5-minute intervals. After fixation and washing, cells were permeabilized with SuperBlock (ThermoFisher Scientific) supplemented with 0.1% Triton-X100 for 1 hour at room temperature. Coverslips were mounted with VECTASHIELD Mounting Medium containing DAPI (4′,6′-diamidino-2-phenylindole; cat# H-1200–10, Vector Laboratories, Inc., Burlingame, CA). Mounted cells were subjected to confocal imaging followed by Mander’s coefficient analyses. 10 cells/mutant were imaged using confocal microscopy. Images were blinded prior to analysis. Blinded images were input into ImageJ and Mander’s coefficients were measured using the JACoP plugin tool.

### qRT-PCR

All cell lysates for qRT-PCR analysis were collected in TRIzol reagent and RNA was isolated using a Direct-zol RNA miniprep kit (Zymo Research, cat# R2051). RNA was converted to cDNA (ThermoFisher, cat# 4368814) using random hexamers following manufacturer’s protocol. cDNA was mixed with respective primers for amplification and performed at the Iowa Institute of Human Genetics. Primers used for experiments can be found in S4 Table. Results were analyzed with the Design and Analysis Software (version 2.6.0). All PCR data is shown as fold change (2^-(ΔΔCt)^) compared to mock infected controls, unless otherwise stated.

### Isolation of primary human monocyte-derived dendritic cells

To isolate peripheral blood mononuclear cells (PBMCs), whole blood was collected from healthy human donors and separated by Ficoll-Paque (Thermo Fisher) gradients. To differentiate cells into dendritic cells, isolated monocytes were maintained in 150-mm dishes in complete RPMI 1640 medium (RPMI 1640 medium supplemented with 10% fetal bovine serum [FBS], nonessential amino acids, 100 U/ml of penicillin, and 100 U/ml of streptomycin) supplemented with human granulocyte-macrophage colony-stimulating factor (GM-CSF, 50 ng/ml) and human interleukin 4 (IL-4; 20 ng/ml) for 5 days. The culture medium was changed every 3 days. Cells were then re-seeded into chamber slides at a density of 1x10^6^ cells/mL into chamber slides (immunocytochemistry) or 6-well plates (immunoblotting) and allowed to further differentiate 2 days prior to infection. DCs were infected with MeV-nYFP at a MOI of 0.1 in Opti-MEM for 3 hours, before the inoculum was removed and replaced with fresh media. 48 hours post infection, cells were fixed with 4% PFA for 15 minutes, washed with PBS, and blocked with Pierce Superblock containing 0.1% Triton X-100 at room temperature for 1 hour. Primary antibodies were incubated overnight in Superblock, including DC-SIGN (Cell Signaling; 13193) 1:100, MeV Nucleoprotein (Sigma-Aldrich; MAB8906) 1:500, and Tom70–594 CoraLite (Proteintech; CL594–14528) 1:200. Secondary antibodies including goat anti-rabbit Alexa Fluor 405 (Invitrogen; A-31556) and donkey anti-mouse Alexa Fluor 647 (Invitrogen; A-31571) were incubated in Superblock at a 1:2000 dilution for 1 hour at room temperature. Chambers were removed from chamber slides and a coverslip was mounted using Vectashield (Vector Laboratories; H-1000–10) and sealed using clear nail polish. Cells were imaged by a Leica Sp8 confocal using a 63x lens.

### Generation of recombinant viruses

YFP fused to triple repeat nuclear localization signal (NLS) was generated as p(+)MV323nYFP as described previously [76,81]. MluI sites flanking the N gene were added using the Quick change system (cat# 200523, Agilent). Fragment between MluI and AgeI was removed from p(+)MV323nYFP and ligated into pCG-PmeIMV323-PmeI. This plasmid was then used for introducing mutations in the N gene. Mutagenesis primers used for experiments can be found in S5 Table. Recombinant viruses containing mutation in N gene at position 13 (MeV-N R13A and MeV-N R13E) were successfully rescued without the need for parental protein complementation. To assess the genomic stability of recombinant MeV, RNA from infected cells were subjected to next-generation sequencing as previously described [76]. Whole genome nucleotide variance analyses showed that the introduced mutations were maintained at >99% indicating genetic stability (S7 Fig).

### Statistics

Statistical tests listed in the figure legends were performed in GraphPad Prism version 10.1.1. Significance was determined by one-way ANOVA with Tukey’s multiple comparisons. Alpha values were set at 0.05. Unless otherwise indicated, numerical data are presented as mean ± standard deviation (SD). Statistical analyses are specified in each figure legend. Significant values are shown by asterisks: *P < 0.05; **P < 0.01; ***P < 0.001; ****P < 0.0001; or ns, not significant.

## Supporting information

S1 Fig MitoVolt assay validation in HAE and H358 cells. (A) H358 cells were infected with MeV-nCFP at an MOI of 0.1 for 4 hrs. At 48 hpi, MitoVolt dye was added and live images were captured. Scale bar = 100 μm. (B) Red/green ratios for panel A are shown. n = 3 biological replicates, 10 fields/replicate. (C and D) Uninfected HAE or H358 cells were vehicle-treated or treated with CCCP for 2 hrs. Following addition of MitoVolt dye, red/green ratios were measured. HAE; n = 3 donors, 2 fields/donor; H358 cells; n = 3 biological replicates, 2 fields/replicate. *p < 0.05, **p < 0.01, ****p < 0.0001.
(TIFF)

S2 Fig cGAS inhibition decreases ISG RNA in H358 cells. H358 cells were mock-infected (grey) or infected with MeV-GFP (MOI 0.1, green). Immediately after infection, vehicle (DMSO) or G140 (10 μM) was applied to media. Treatments were replaced every 24 hrs. After 3 dpi, RNA was extracted and subjected to ISG expression analysis. n = 3. (F) HEK293T, H358, or HeLa cells were transfected with G3-YSD without cGAS inhibitor, treated with cGAS inhibitor only, or transfected with G3-YSD and provided cGAS inhibitor. 18 hrs post-transfection, cells were collected in TRIzol and subjected to RNA extraction. Level of IFN-β was measured as an output for cGAS inhibition or activation. (G) H358 cells were infected with MeV-GFP (MOI 0.1) and treated with vehicle, G140, or transfected with G3-YSD. Images were captured at 48 hpi. Scale bar = 500 μm. (H) H358 cells from panel G were harvested for titer analysis, n = 3. (I) H358 cells were infected with MeV-YFP, PIV5-mCherry, or RSV-GFP (MOI 0.1) and treated immediately after infection with G140 (10 μM, green bars) or vehicle (DMSO, gray bars). Treatments were replaced every 24 hrs. Images were captured at 24, 48, and 72 hpi. Relative fluorescence units (RFU) were quantified via ImageJ, n = 6. **p < 0.01, ***p < .001, ****p < 0.0001.
(TIFF)

S3 Fig Inhibition of mtDNA replication in H358 cells increases MeV infection. (A) H358 cells were treated with vehicle (DMSO) or ddC (100 μM) 48 hrs pre-infection. After 48 hrs of treatment, cells were infected with MeV-GFP (MOI 0.1). Treatments were replaced every 24 hrs during the experiment. (B) Syncytia sizes were measured via ImageJ 24 and 48 hpi. (C) 48 hrs post-treatment with vehicle or ddC, RNA was extracted from H358 cells. mtDNA depletion was confirmed by RT-qPCR of the D-loop gene. (D) H358 cells were infected with MeV-GFP and treated with either vehicle or ddC. Cells were harvested at 48 hpi and subjected to titer analysis. (E-I) H358 cells were treated with vehicle or ddC 48 hrs pre-infection. After 48 hrs, the cells were either infected with MeV-GFP (MOI 0.1) or mock-infected. RNA was extracted 3 dpi and subjected to ISG expression analysis. n = 3 biological replicates. ns, not significant, *p < 0.05, **p < 0.01, ***p < .001, ****p < 0.0001.
(TIFF)

S4 Fig MeV N is enriched in the mitochondrial fraction of H358 cells. (A) H358 cells were mock- or MeV-infected. WCLs, cytoplasmic, and mitochondrial fractions were isolated and immunoblotted for vinculin (cytoplasmic protein), NDUFS1 (mitochondrial protein), and MeV N protein. (B) MitoFates MLS probability was determined for vinculin, NDUFS1, and MeV N protein. n.d., not detected. (C) Dendritic cells (DCs) were infected with MeV-nYFP (pseudocolored green, MOI 0.1) and imaged at 2 dpi. Scale bar = 20 mm. Indicated virus co-stained for N protein (white), Tom70 (red), and DC-SIGN (blue). Arrows indicate areas of co-localization. (D) The Mander’s coefficient between N and Tom70 from panel C is indicated. (E) Dendritic cells were mock- or MeV-infected. WCLs, cytoplasmic, and mitochondrial fractions were isolated and immunoblotted for vinculin (cytoplasmic protein), NDUFS1 (mitochondrial protein), and MeV N protein.
(TIFF)

S5 Fig MitoFates predictions of the impact of N protein mutations on mitochondria localization. N70-GFP plasmid sequences containing the indicated mutations (top) or native WT MeV N sequence containing the indicated mutations (bottom) were input into MitoFates.
(TIFF)

S6 Fig MeV N mutants colocalize with mitochondria. (A) Here the images from Fig 7E–7G are separated into individual channels. H358 cells were infected with MeV-nYFP (pseudocolored green, MOI 0.1) with the indicated amino acid substitution in the N protein and imaged at 2 dpi. Scale bar = 4 mm. Indicated virus was co-stained for N protein (white) and Tom70 (red). Arrows indicate areas of co-localization. (B) Mander’s coefficient analysis of co-localization is shown. Each point indicates one cell from 3 independent experiments. * p < 0.05; ns, not significant.
(TIFF)

S7 Fig Next-generation sequencing analyses of parental and mutant MeV stocks documenting the stability of the introduced mutations. The percentage of reads corresponding to the introduced mutations is indicated. *x*-axis: MeV genome. *y*-axis: number of reads per nucleotide. Blue lines represent positive strand reads (MeV mRNA) and orange lines represent negative strand reads (MeV genomic RNA). For all viruses, positive strand reads (blue lines) decreased progressively with the distance of the six genes from the 3’ end of the genome. This reflects an expected transcriptional attenuation. For all viruses, negative strand reads (orange lines) were detected at constant levels, indicating the absence of small defective genomes.
(TIFF)

S1 Table Accession numbers for proteins used for MitoFates analyses. (DOCX)

S2 Table Accession numbers for MeV-specific proteins used for MitoFates analyses. (DOCX)

S3 Table Forward and reverse primers used for MeV-N70-GFP mutant generation. (DOCX)

S4 Table Forward and reverse primers used for qRT-PCR analyses. ^#^The MeV L-trailer primers were used for genomic specific amplification.
(DOCX)

S5 Table Forward and reverse primers used for viral genome modification. (DOCX)
