# Supplementary figures and images for: Mitochondrial targeting by measles virus nucleoprotein modulates viral spread in human airway epithelium
Source: PLoS Pathog. 2025 Nov 20;21(11):e1013713. doi: 10.1371/journal.ppat.1013713 (PMC12646431; doi:10.1371/journal.ppat.1013713)

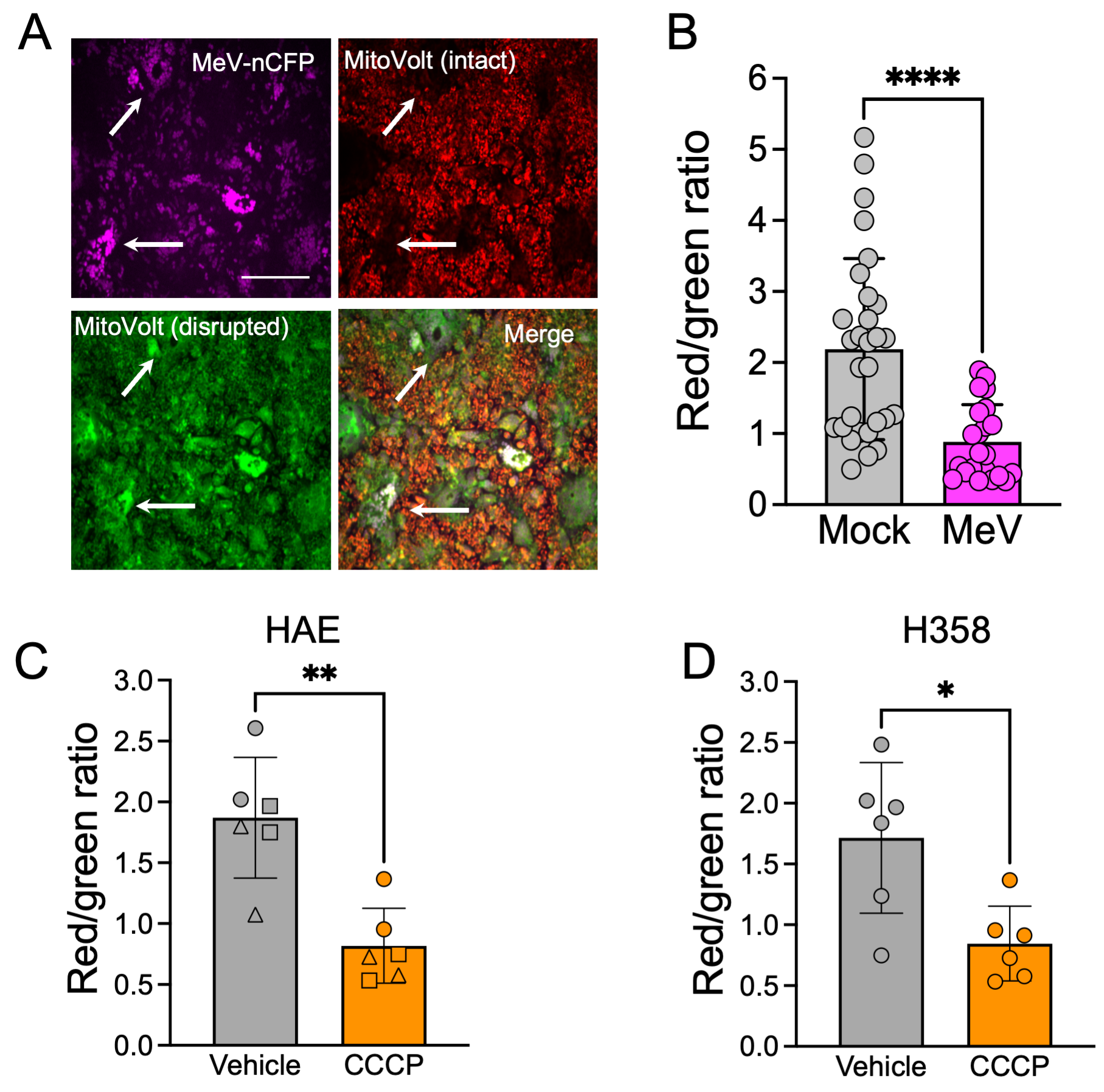

Supplement: S1 Fig — (A) H358 cells were infected with MeV-nCFP at an MOI of 0.1 for 4 hrs. At 48 hpi, MitoVolt dye was added and live images were captured. Scale bar = 100 μm. (B) Red/green ratios for panel A are shown. n = 3 biological replicates, 10 fields/replicate. (C and D) Uninfected HAE or H358 cells were vehicle-treated or treated with CCCP for 2 hrs. Following addition of MitoVolt dye, red/green ratios were measured. HAE; n = 3 donors, 2 fields/donor; H358 cells; n = 3 biological replicates, 2 fields/replicate. *p < 0.05, **p < 0.01, ****p < 0.0001. (TIFF) [file ppat.1013713.s001.tiff]

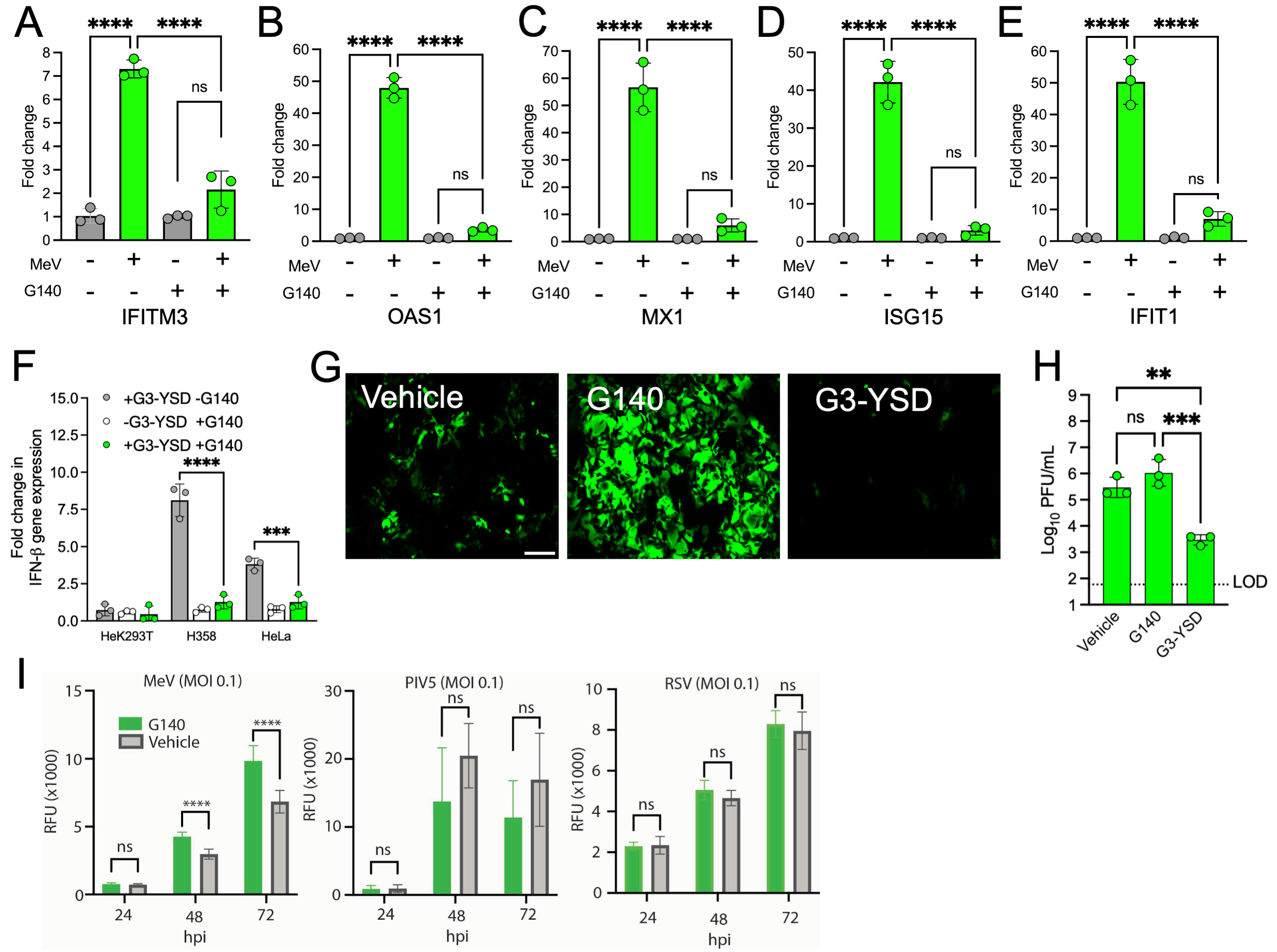

Supplement: S2 Fig — H358 cells were mock-infected (grey) or infected with MeV-GFP (MOI 0.1, green). Immediately after infection, vehicle (DMSO) or G140 (10 μM) was applied to media. Treatments were replaced every 24 hrs. After 3 dpi, RNA was extracted and subjected to ISG expression analysis. n = 3. (F) HEK293T, H358, or HeLa cells were transfected with G3-YSD without cGAS inhibitor, treated with cGAS inhibitor only, or transfected with G3-YSD and provided cGAS inhibitor. 18 hrs post-transfection, cells were collected in TRIzol and subjected to RNA extraction. Level of IFN-β was measured as an output for cGAS inhibition or activation. (G) H358 cells were infected with MeV-GFP (MOI 0.1) and treated with vehicle, G140, or transfected with G3-YSD. Images were captured at 48 hpi. Scale bar = 500 μm. (H) H358 cells from panel G were harvested for titer analysis, n = 3. (I) H358 cells were infected with MeV-YFP, PIV5-mCherry, or RSV-GFP (MOI 0.1) and treated immediately after infection with G140 (10 μM, green bars) or vehicle (DMSO, gray bars). Treatments were replaced every 24 hrs. Images were captured at 24, 48, and 72 hpi. Relative fluorescence units (RFU) were quantified via ImageJ, n = 6. **p < 0.01, ***p < .001, ****p < 0.0001. (TIFF) [file ppat.1013713.s002.tiff]

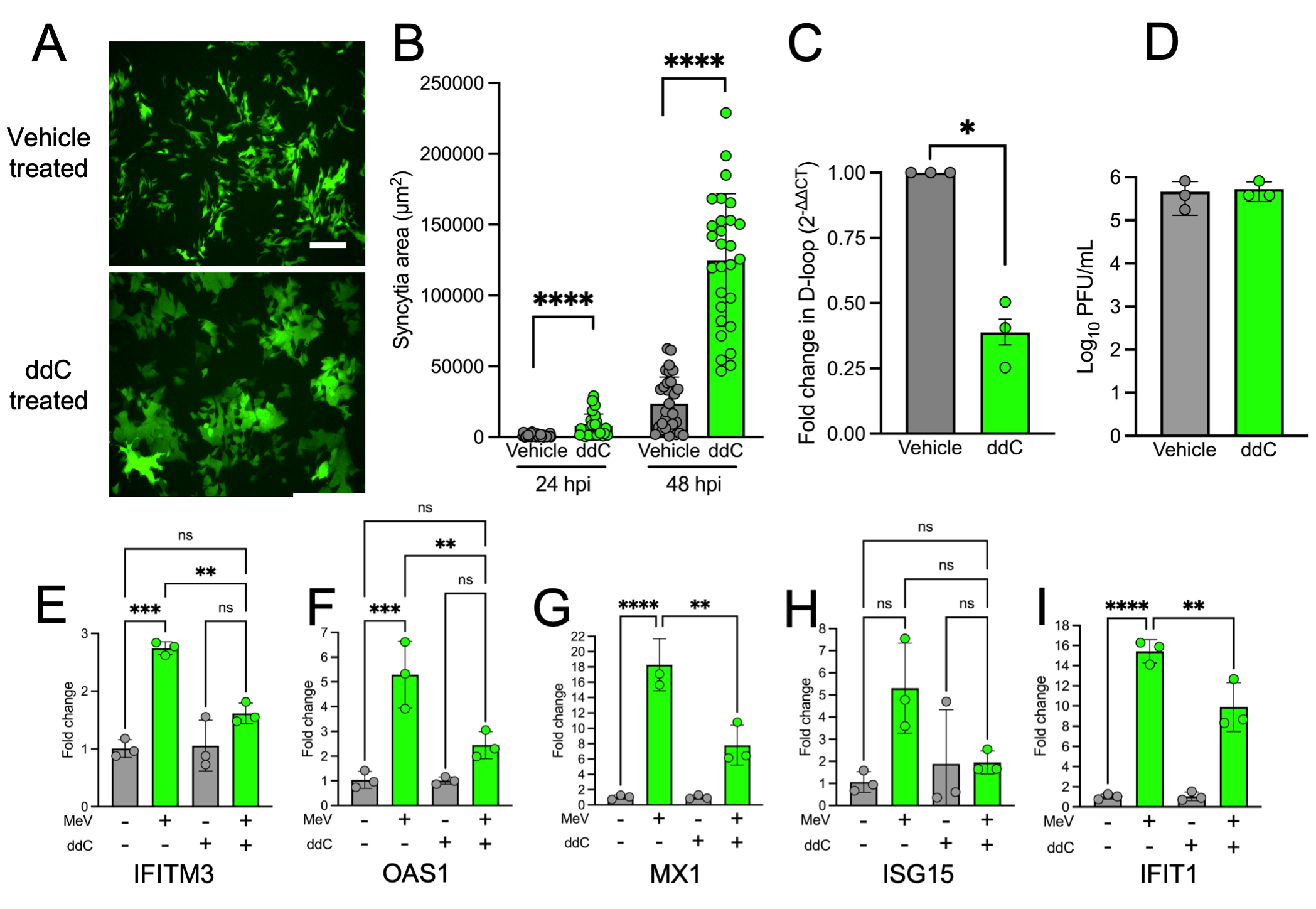

Supplement: S3 Fig — (A) H358 cells were treated with vehicle (DMSO) or ddC (100 μM) 48 hrs pre-infection. After 48 hrs of treatment, cells were infected with MeV-GFP (MOI 0.1). Treatments were replaced every 24 hrs during the experiment. (B) Syncytia sizes were measured via ImageJ 24 and 48 hpi. (C) 48 hrs post-treatment with vehicle or ddC, RNA was extracted from H358 cells. mtDNA depletion was confirmed by RT-qPCR of the D-loop gene. (D) H358 cells were infected with MeV-GFP and treated with either vehicle or ddC. Cells were harvested at 48 hpi and subjected to titer analysis. (E-I) H358 cells were treated with vehicle or ddC 48 hrs pre-infection. After 48 hrs, the cells were either infected with MeV-GFP (MOI 0.1) or mock-infected. RNA was extracted 3 dpi and subjected to ISG expression analysis. n = 3 biological replicates. ns, not significant, *p < 0.05, **p < 0.01, ***p < .001, ****p < 0.0001. (TIFF) [file ppat.1013713.s003.tiff]

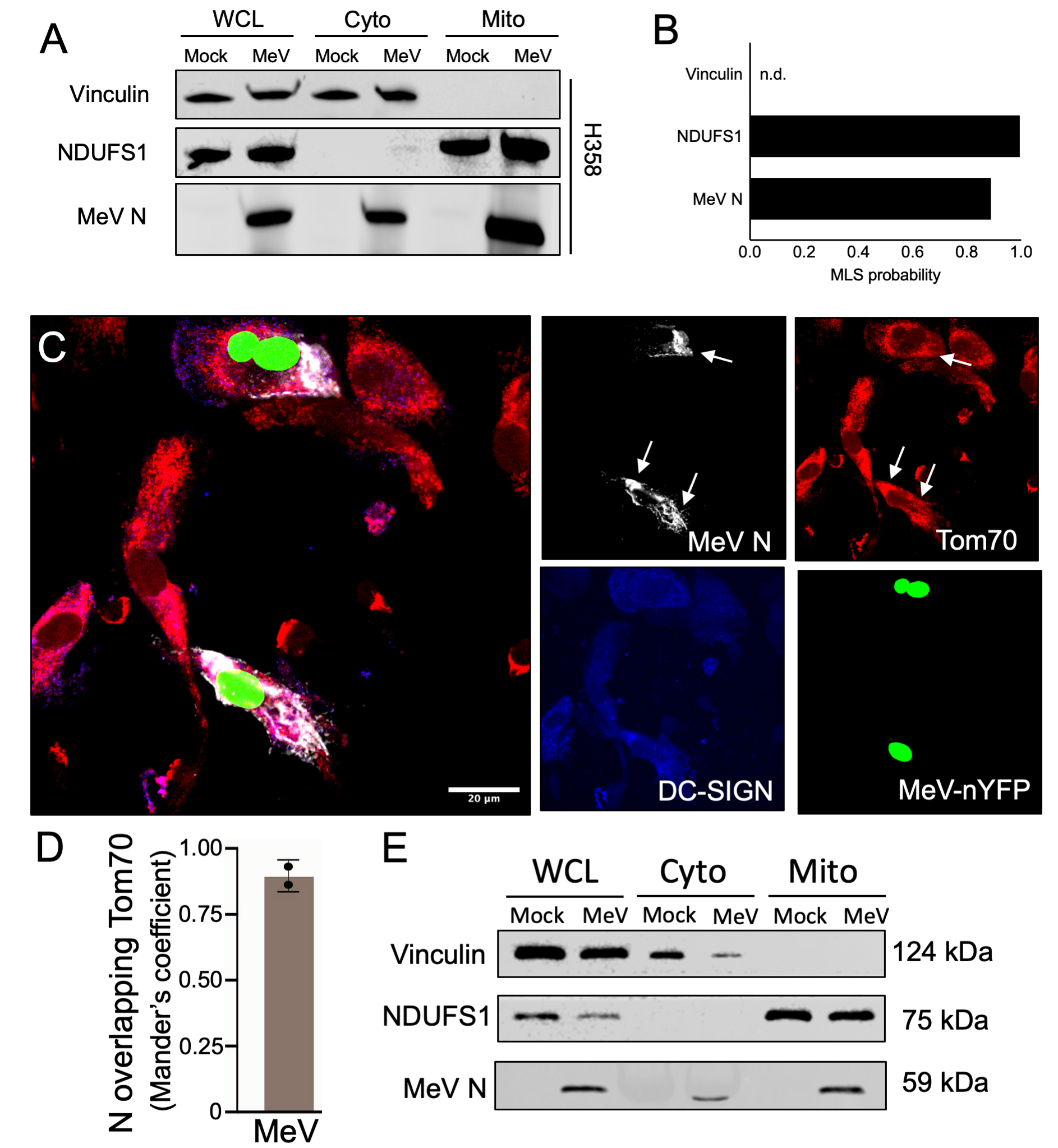

Supplement: S4 Fig — (A) H358 cells were mock- or MeV-infected. WCLs, cytoplasmic, and mitochondrial fractions were isolated and immunoblotted for vinculin (cytoplasmic protein), NDUFS1 (mitochondrial protein), and MeV N protein. (B) MitoFates MLS probability was determined for vinculin, NDUFS1, and MeV N protein. n.d., not detected. (C) Dendritic cells (DCs) were infected with MeV-nYFP (pseudocolored green, MOI 0.1) and imaged at 2 dpi. Scale bar = 20 mm. Indicated virus co-stained for N protein (white), Tom70 (red), and DC-SIGN (blue). Arrows indicate areas of co-localization. (D) The Mander’s coefficient between N and Tom70 from panel C is indicated. (E) Dendritic cells were mock- or MeV-infected. WCLs, cytoplasmic, and mitochondrial fractions were isolated and immunoblotted for vinculin (cytoplasmic protein), NDUFS1 (mitochondrial protein), and MeV N protein. (TIFF) [file ppat.1013713.s004.tiff]

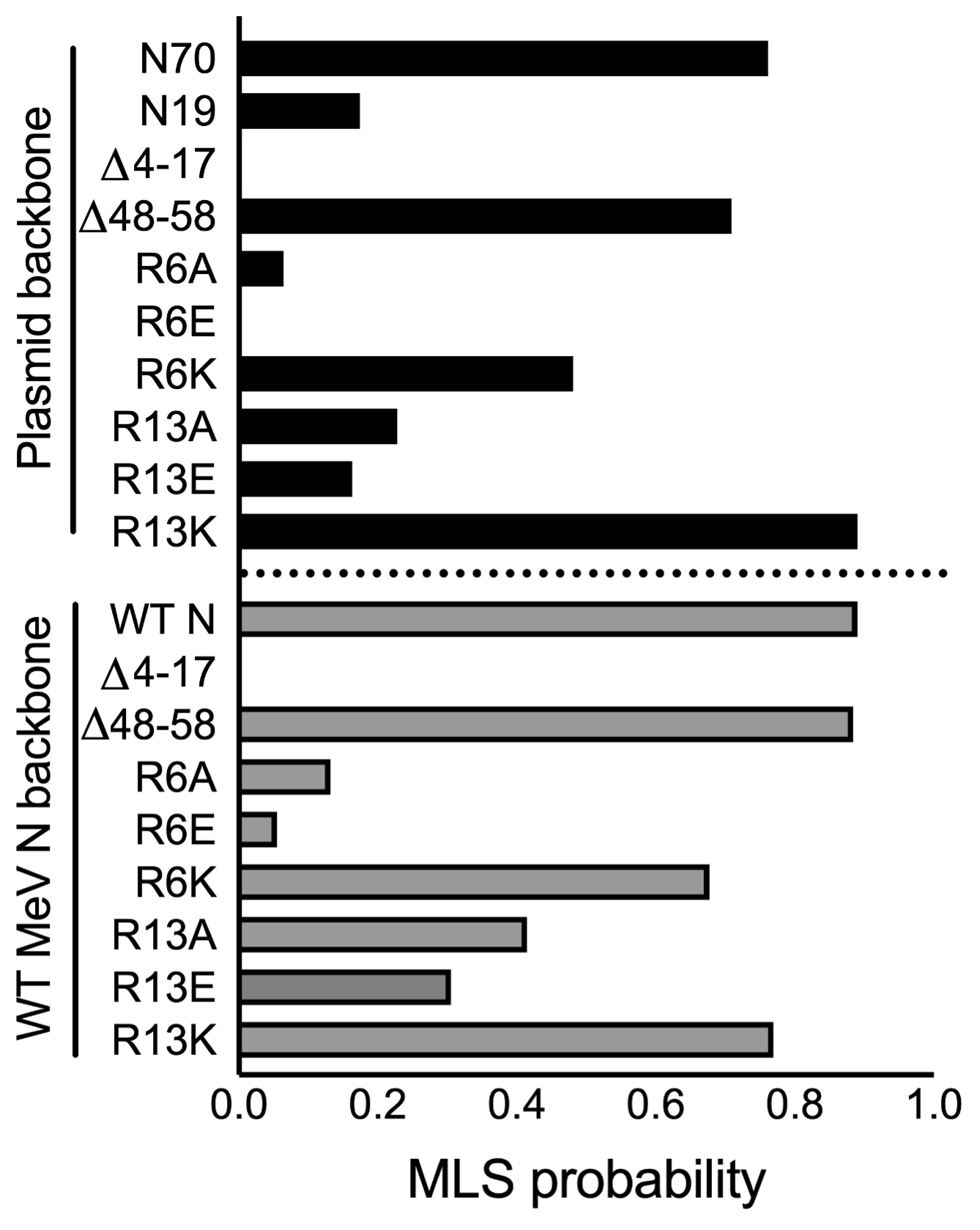

Supplement: S5 Fig — N70-GFP plasmid sequences containing the indicated mutations (top) or native WT MeV N sequence containing the indicated mutations (bottom) were input into MitoFates. (TIFF) [file ppat.1013713.s005.tiff]

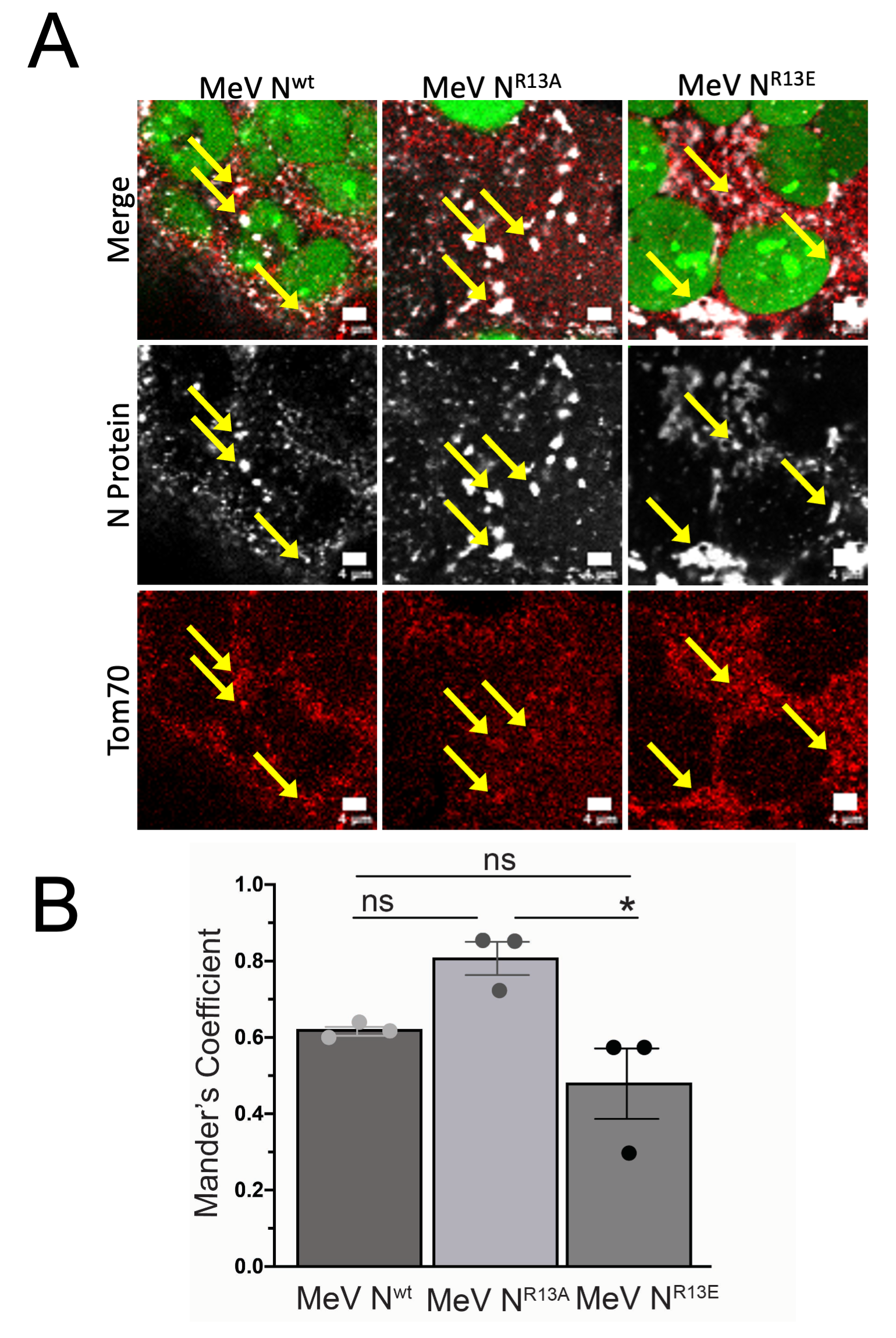

Supplement: S6 Fig — (A) Here the images from Fig 7E–7G are separated into individual channels. H358 cells were infected with MeV-nYFP (pseudocolored green, MOI 0.1) with the indicated amino acid substitution in the N protein and imaged at 2 dpi. Scale bar = 4 mm. Indicated virus was co-stained for N protein (white) and Tom70 (red). Arrows indicate areas of co-localization. (B) Mander’s coefficient analysis of co-localization is shown. Each point indicates one cell from 3 independent experiments. * p < 0.05; ns, not significant. (TIFF) [file ppat.1013713.s006.tiff]

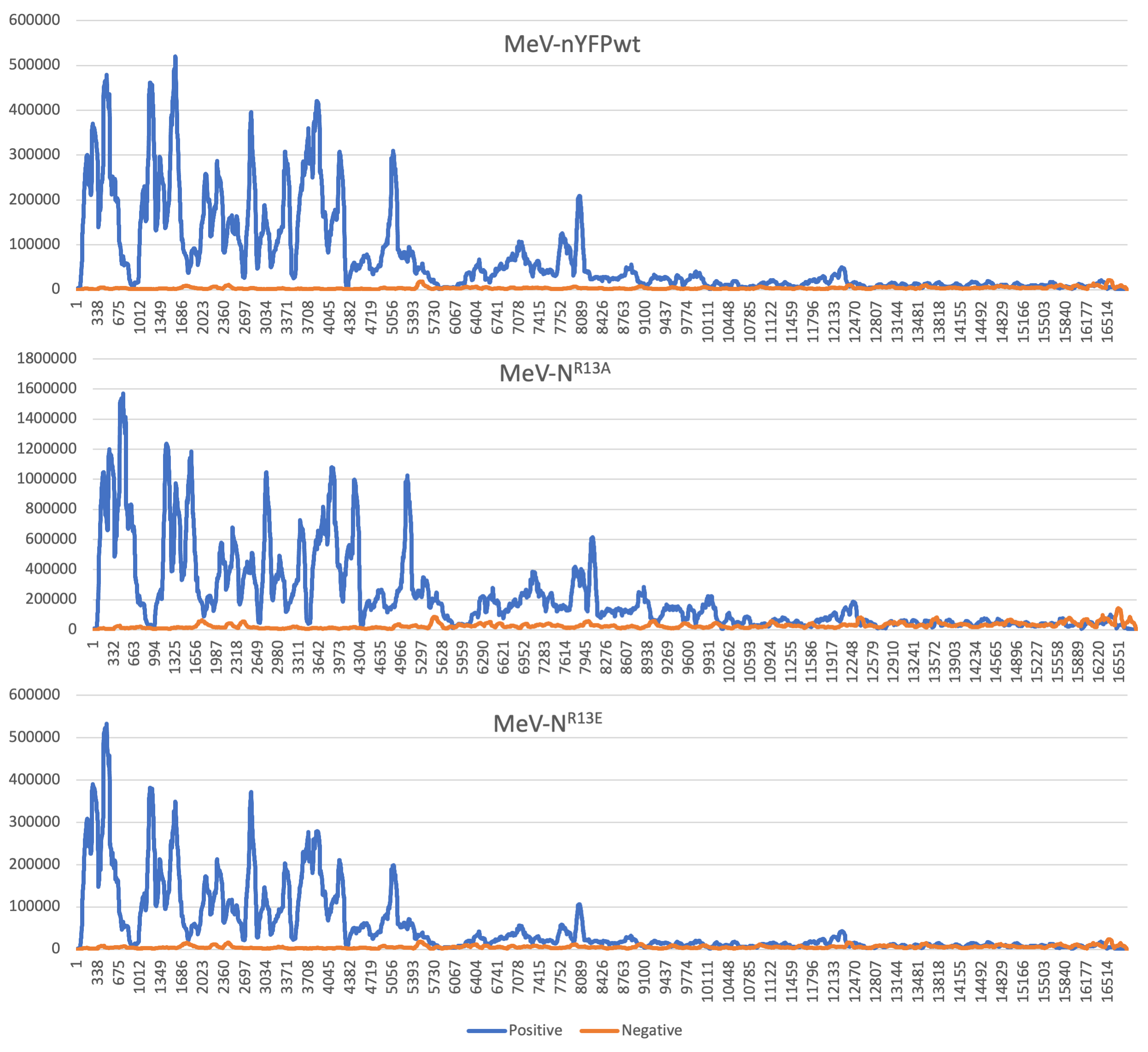

Supplement: S7 Fig — The percentage of reads corresponding to the introduced mutations is indicated. x-axis: MeV genome. y-axis: number of reads per nucleotide. Blue lines represent positive strand reads (MeV mRNA) and orange lines represent negative strand reads (MeV genomic RNA). For all viruses, positive strand reads (blue lines) decreased progressively with the distance of the six genes from the 3’ end of the genome. This reflects an expected transcriptional attenuation. For all viruses, negative strand reads (orange lines) were detected at constant levels, indicating the absence of small defective genomes. (TIFF) [file ppat.1013713.s007.tiff]
