# Supplementary material for: Mitochondrial targeting by measles virus nucleoprotein modulates viral spread in human airway epithelium
Source: PLoS Pathog. 2025 Nov 20;21(11):e1013713. doi: 10.1371/journal.ppat.1013713 (PMC12646431; doi:10.1371/journal.ppat.1013713)
Supplement: S1 Table — (DOCX) [file ppat.1013713.s008.docx]

| **Protein accession numbers** | | | |
| --- | --- | --- | --- |
| Category | Species | | Accession # |
| N protein of other –ssRNA viruses | *Orthopneumovirus hominis* (RSV) | | NC_038235 |
|  | *Lyssavirus rabies* | | NC_001542 |
|  | *Orthobornavirus avisaquaticae* | | NC_030691 |
|  | *Orthoebolavirus zairense* | | NC_002549 |
| Morbillivirus-specific N protein | *M. ceti* | | ON513035 |
|  | *M. hominis* | | NC_001498 |
|  | *M. pecoris* | | NC_006296 |
|  | *M. caprinae* | | NC_006383 |
|  | *M. felis* | | NC_039196 |
|  | *M. canis* | | AF378705 |
|  | *M. phocae* | | NC_028249 |
| N proteins from other MeV isolates | Genotype D4 | 0.9 MLS prob | MF449469 |
|  | Genotype C2 | 0.9 MLS prob | MG912589 |
|  | Genotype D8 | 0.9 MLS prob | OR290099 |
|  | Genotype D3 | 0.9 MLS prob | OQ096316 |
|  | Schwarz | 0.9 MLS prob | AF266291 |
|  | Moraten | 0.9 MLS prob | AF266287 |
|  | Edmonston (vaccine) | 0.9 MLS prob | AF266290.1 |
| N proteins from other paramyxoviruses | *Orthorubulavirus* | | NC_006430 |
|  | *Henipavirus* | | NC_001906 |
|  | *Synodonvirus* | | NC_075439 |
|  | *Paraavulavirus* | | NC_025373 |
| Control proteins for study | hMSRB2 | | KAI4075455.1 |
|  | GRP78 | | AAI12964.1 |
|  | GFP | | ANA76508.1 |
|  | NDUFS1 | | NP_001186910.1 |
|  | Vinculin | | AAA61283 |
