# Supplementary material for: Mitochondrial targeting by measles virus nucleoprotein modulates viral spread in human airway epithelium
Source: PLoS Pathog. 2025 Nov 20;21(11):e1013713. doi: 10.1371/journal.ppat.1013713 (PMC12646431; doi:10.1371/journal.ppat.1013713)
Supplement: S2 Table — (DOCX) [file ppat.1013713.s009.docx]

| **Protein accession numbers** | |
| --- | --- |
| Protein ID | Accession # |
| N | NC_038235 |
| P | NP_056919.1 |
| V | YP_003873249.2 |
| C | NP_056920.1 |
| M | NP_056921.1 |
| F | Np_056922.1 |
| H | NP_056923.1 |
| L | NP_056924.1 |
