# Supplementary material for: Mitochondrial targeting by measles virus nucleoprotein modulates viral spread in human airway epithelium
Source: PLoS Pathog. 2025 Nov 20;21(11):e1013713. doi: 10.1371/journal.ppat.1013713 (PMC12646431; doi:10.1371/journal.ppat.1013713)
Supplement: S3 Table — (DOCX) [file ppat.1013713.s010.docx]

| **Target mutation** | **Direction** | **Sequence** |
| --- | --- | --- |
| R6A | Fwd | ACTTTTGCCGAGCTTAGCATTGTTCAAAAGAAAC |
|  | Rev | AAGCTCGCCAAAAGTGTGGCCATGGTGG |
| R6E | Fwd | CACACTTTTGGAAAGCTTAGCATTGTTC |
|  | Rev | GCCATTTGTTAGTTTGG |
| R6K | Fwd | ACTTTTGAAAAGCTTAGCATTGTTCAAAAGAAAC |
|  | Rev | AAGCTCTTCAAAAGTGTGGCCATGGTGG |
| R13A | Fwd | GTTCAAAGCAAACAAGGACAAACCACCCATTAC |
|  | Rev | TTGTTTGCTTTGAACAATGCTAAGCTCCTC |
| R13E | Fwd | ATTGTTCAAAGAAAACAAGGACAAACC |
|  | Rev | GCTAAGCTCCTCAAAAG |
| R13K | Fwd | GTTCAAAAAGAACAAGGACAAACCACCCATTAC |
|  | Rev | TTGTTCTTTTTGAACAATGCTAAGCTCCTC |
| △4-17 | Fwd | CCACCCATTACATCAGGATCCGGT |
|  | Rev | TGTGGCCATGGTGGCG |
| N19 | Fwd | CACCCATTACATCAGGATCCGGT |
|  | Rev | GGGTGGTTTGTCCTTGTTTC |
| N32 | Fwd | CACCCATTACATCAGGATCCGGT |
|  | Rev | AGCGGCCGCATGAGCAAGGG |
