# Supplementary material for: Mitochondrial targeting by measles virus nucleoprotein modulates viral spread in human airway epithelium
Source: PLoS Pathog. 2025 Nov 20;21(11):e1013713. doi: 10.1371/journal.ppat.1013713 (PMC12646431; doi:10.1371/journal.ppat.1013713)
Supplement: S4 Table — #The MeV L-trailer primers were used for genomic specific amplification. (DOCX) [file ppat.1013713.s011.docx]

| **Target gene** | **Direction** | **Sequence** |
| --- | --- | --- |
| 18S rRNA | Fwd | TGTGCCGCTAGAGGTGAAATT |
|  | Rev | TGGCAAATGCTTTCGCTTT |
| MeV N RNA | Fwd | ATTGACACTGCAACGGAGTC |
|  | Rev | GCCTTGTTCTTCCGAGATTC |
| MeV L-trailer^#^ | Fwd | TGAACTCCGGAACCCTAATC |
|  | Rev | AAACCTGGGAATAGAAACTTCG |
| Mitochondrial D-loop | Fwd | GACCACCATCCTCCGTGAAATC |
|  | Rev | TCACTTTAGCTACCCCCAAGTGTTA |
| IFN-β | Fwd | GTTGAGAACCTCCTGGCTAATG |
|  | Rev | GGTAATGCAGAATCCTCCCATAATA |
| IFITM3 | Fwd | TGAAGTCTAGGGACAGGAAGA |
|  | Rev | CATGAGGATGCCCAGAATCA |
| IFIT1 | Fwd | CCAGGTCACCAGACTCCTCA |
|  | Rev | GGAATACACAACCTACTAGCC |
| OAS1 | Fwd | TGAGCTCCTGGATTCTGCTGAC |
|  | Rev | TGGCATTCAGAGGATGGTGCAG |
| MX1 | Fwd | ACCTGGTCCTGGCAGTAGACAATC |
|  | Rev | TAACCTCCACAGAACCGCCAAGTC |
| ISG15 | Fwd | GAGAGGCAGCGAACTCATCT |
|  | Rev | CTTCAGCTCTGACACCGACA |
