# Supplementary material for: Mitochondrial targeting by measles virus nucleoprotein modulates viral spread in human airway epithelium
Source: PLoS Pathog. 2025 Nov 20;21(11):e1013713. doi: 10.1371/journal.ppat.1013713 (PMC12646431; doi:10.1371/journal.ppat.1013713)
Supplement: S5 Table — (DOCX) [file ppat.1013713.s012.docx]

| **Target mutation** | **Direction** | **Sequence** |
| --- | --- | --- |
| R13A | Fwd | ACTTTTGAGGAGCTTAGCATTGTTCAAA**GC**AAACAAGGACAAACCACCCATTACATC |
|  | Rev | GATGTAATGGGTGGTTTGTCCTTGTTT**GC**TTTGAACAATGCTAAGCTCCTCAAAAGT |
| R13E | Fwd | TGAGGAGCTTAGCATTGTTCAAA**GA**AAACAAGGACAAACCACCCATTAC |
|  | Rev | GTAATGGGTGGTTTGTCCTTGTTT**TC**TTTGAACAATGCTAAGCTCCTCA |
